# Identification of Interleukin1β as an Amplifier of Interferon alpha-induced Antiviral Responses

**DOI:** 10.1371/journal.ppat.1008461

**Published:** 2020-10-01

**Authors:** Katharina Robichon, Tim Maiwald, Marcel Schilling, Annette Schneider, Joschka Willemsen, Florian Salopiata, Melissa Teusel, Clemens Kreutz, Christian Ehlting, Jun Huang, Sajib Chakraborty, Xiaoyun Huang, Georg Damm, Daniel Seehofer, Philipp A. Lang, Johannes G. Bode, Marco Binder, Ralf Bartenschlager, Jens Timmer, Ursula Klingmüller

**Affiliations:** 1 Division Systems Biology of Signal Transduction, German Cancer Research Center (DKFZ), Heidelberg, Germany; 2 Institute for Physics, University of Freiburg, Germany; 3 FDM—Freiburg Center for Data Analysis and Modeling, University of Freiburg, Freiburg, Germany; 4 Research Group "Dynamics of Early Viral Infection and the Innate Antiviral Response“, Division Virus-Associated Carcinogenesis, German Cancer Research Center (DKFZ), Heidelberg, Germany; 5 Institute of Medical Biometry and Statistics, Faculty of Medicine and Medical Center, University of Freiburg, Freiburg, Germany; 6 Department of Gastroenterology, Hepatology and Infectious Diseases, University Hospital, Medical Faculty, Heinrich-Heine-University of Düsseldorf, Germany; 7 Department of Molecular Medicine II, University Hospital, Medical Faculty, Heinrich-Heine-University of Düsseldorf, Germany; 8 Department of Biochemistry and Molecular Biology, University of Dhaka, Dhaka, Bangladesh; 9 Department of Hepatobiliary Surgery and Visceral Transplantation, University of Leipzig, Leipzig, Germany and Department of General-, Visceral- and Transplantation Surgery, Charité University Medicine Berlin, Berlin, Germany; 10 Department of Infectious Diseases, Molecular Virology, University of Heidelberg, Heidelberg, Germany; 11 Signalling Research Centres BIOSS and CIBSS, University of Freiburg, Freiburg, Germany; Cleveland Clinic Florida, UNITED STATES

## Abstract

The induction of an interferon-mediated response is the first line of defense against pathogens such as viruses. Yet, the dynamics and extent of interferon alpha (IFNα)-induced antiviral genes vary remarkably and comprise three expression clusters: early, intermediate and late. By mathematical modeling based on time-resolved quantitative data, we identified mRNA stability as well as a negative regulatory loop as key mechanisms endogenously controlling the expression dynamics of IFNα-induced antiviral genes in hepatocytes. Guided by the mathematical model, we uncovered that this regulatory loop is mediated by the transcription factor IRF2 and showed that knock-down of IRF2 results in enhanced expression of early, intermediate and late IFNα-induced antiviral genes. Co-stimulation experiments with different pro-inflammatory cytokines revealed that this amplified expression dynamics of the early, intermediate and late IFNα-induced antiviral genes can also be achieved by co-application of IFNα and interleukin1 beta (IL1β). Consistently, we found that IL1β enhances IFNα-mediated repression of viral replication. Conversely, we observed that in IL1β receptor knock-out mice replication of viruses sensitive to IFNα is increased. Thus, IL1β is capable to potentiate IFNα-induced antiviral responses and could be exploited to improve antiviral therapies.

## Author summary

Innate immune responses contribute to the control of viral infections and the induction of interferon alpha (IFNα)-mediated antiviral responses is an important component. However, IFNα induces a multitude of antiviral response genes and the expression dynamics of these genes can be classified as early, intermediate and late. Here we show, based on a mathematical modeling approach, that mRNA stability as well as the negative regulator IRF2 control the expression dynamics of IFNα-induced antiviral genes. Knock-down of IRF2 resulted in the amplified IFNα-mediated induction of the antiviral genes and this amplified expression of antiviral genes could be functionally mimicked by co-stimulation with IFNα and IL1β. We observed that co-stimulation with IFNα and IL1β enhanced the repression of virus replication and that knock-out of the IL1 receptor in mice resulted in increased replication of a virus sensitive to IFNα. In sum, our studies identified IL1β as an important amplifier of IFNα-induced antiviral responses.

## Introduction

Cytokines such as interferons (IFNs) are important regulators of the innate immune system, the first line of defense against microbial infection. IFNs induce in a highly dynamic process the expression of several classes of IFN-stimulated genes. The encoded proteins of these genes fulfill a variety of tasks including the clearance of viruses. To ensure effectiveness of the response and to prevent damage, the process has to be tightly controlled, which is achieved through several positive and negative feedback loops [1]. Due to the non-linearity of the underlying reactions the impact of alterations on a potential outcome is difficult to predict. IFNs such as interferon alpha (IFNα) are widely applied therapeutic agents and therefore strategies to strengthen IFN-induced responses are of major interest. However, this requires a more quantitative understanding of the interrelations between the IFN signaling pathway components and the expression of IFN-stimulated genes (ISGs) as well as insights into mechanisms shaping the response to IFNs.

A well-studied IFN-induced response is the antiviral response elicited for example by major hepatotropic RNA viruses such as the human pathogen hepatitis C virus (HCV) and the murine pathogen lymphocytic choriomeningitis virus (LCMV). Upon infection, the viral RNA is sensed by specific cellular pattern recognition receptors (PRR) that trigger the expression of interferons (IFNs) and induce expression of antiviral genes as first line of defense [2]. However, viruses can evade the antiviral response by antagonizing the induction of the effector pathways of the IFN system and establish a persistent infection. Therefore, it would be highly beneficial to identify mechanisms to enhance the IFN-induced antiviral response to reduce virus spread and improve viral clearance.

The major signal transduction pathway activated in response to type I IFNs such as IFNα is the JAK/STAT pathway [3]. Regulation of the dynamics of the JAK/STAT pathway activation and the expression of IFN-stimulated genes are important to mount an effective IFN response and to maintain cellular homeostasis. The IFNα-induced signaling pathway comprises complex negative feedback loops consisting of suppressor of cytokine signaling 1 (SOCS1) and ubiquitin-specific peptidase 18 (USP18) that jointly determine signal attenuation. In contrast, interferon regulatory factor 9 (IRF9) acts as a positive regulator of IFNα signaling. By dynamic pathway modeling it was shown that an upregulation of IRF9 can enhance the expression of ISGs [4]. Further, it was shown that the extent and duration of the expression of antiviral genes positively correlates with a reduced virus load [5] and the specific expression profiles of antiviral genes appear to be critical for shifting the balance from viral persistence to viral clearance. Therefore, the modulation of feedback loops might be harnessed to increase and prolong the duration of the IFN response and thereby contribute to improved viral clearance.

IFNα was not only shown to activate the classical JAK-STAT1 pathway, but recent publications have also reported an activation of STAT3 after IFNα treatment [6]. For example, Su et al. showed a phosphorylation of STAT3 after IFNα treatment in RAMOS cells [7] and IFNα treatment led to an increase of STAT3 phosphorylation in primary healthy dendritic cells [8] as well as B cells [9]. The activation as well as the molecular abundance of the different STAT molecules promotes the formation of different hetero- and homodimer pairs, resulting in different expression dynamics of the ISGs [10].

In addition to type I IFNs, pro-inflammatory cytokines such as interleukin 6 (IL6), interleukin-1beta (IL1β) and IFN gamma (IFNγ) [11] can contribute to the activation of an anti-microbial response. Binding of IL1β to the type I IL1 receptor (IL1R1) that is expressed on different cell types including hepatocytes results in the activation of different downstream signaling pathways. While the main pathways activated by IL1β are p38 and NFκB [12], there is evidence that IL1β can also activate STAT3 [13]. IL1β was reported to induce the protein-protein interaction between STAT3 and NFκB in hepatocytes as well as DNA binding of this complex [14], which might be involved in facilitating the recently reported NFκB-assisted DNA loading of STAT3 during the acute phase response [15]. An interplay between IFNα and IL1β has been observed previously. On the one hand, in liver samples of chronic hepatitis C patients elevated levels of IFNβ and IL1β were observed [16]. On the other hand, it was reported that IFNα and IFNβ suppress IL1β maturation in bone marrow-derived macrophages [17] and that IL1β limits excessive type I IFN production through the induction of eicosanoids [18]. Co-treatment with IFNα and IL1β resulted in higher and more sustained STAT1 phosphorylation in Huh7 cells [19]. Thus, the physiological relevance and the underlying mechanism of a potential cross-talk between type I IFN-induced signaling and IL1β remains unknown.

Here we employ a systems biology approach that combines time-resolved quantitative experimental data and mathematical modeling. We show that mRNA stability as well as IRF2 as a negative feedback loop critically shape the distinct expression dynamics of the early, intermediate and late IFNα-induced genes. Importantly, we uncover that knockdown of IRF2 and co-stimulation with IL1β can boost the IFNα-induced antiviral gene response.

## Results

### Distinct dynamics of IFNα-induced gene expression

**Fig 1:**
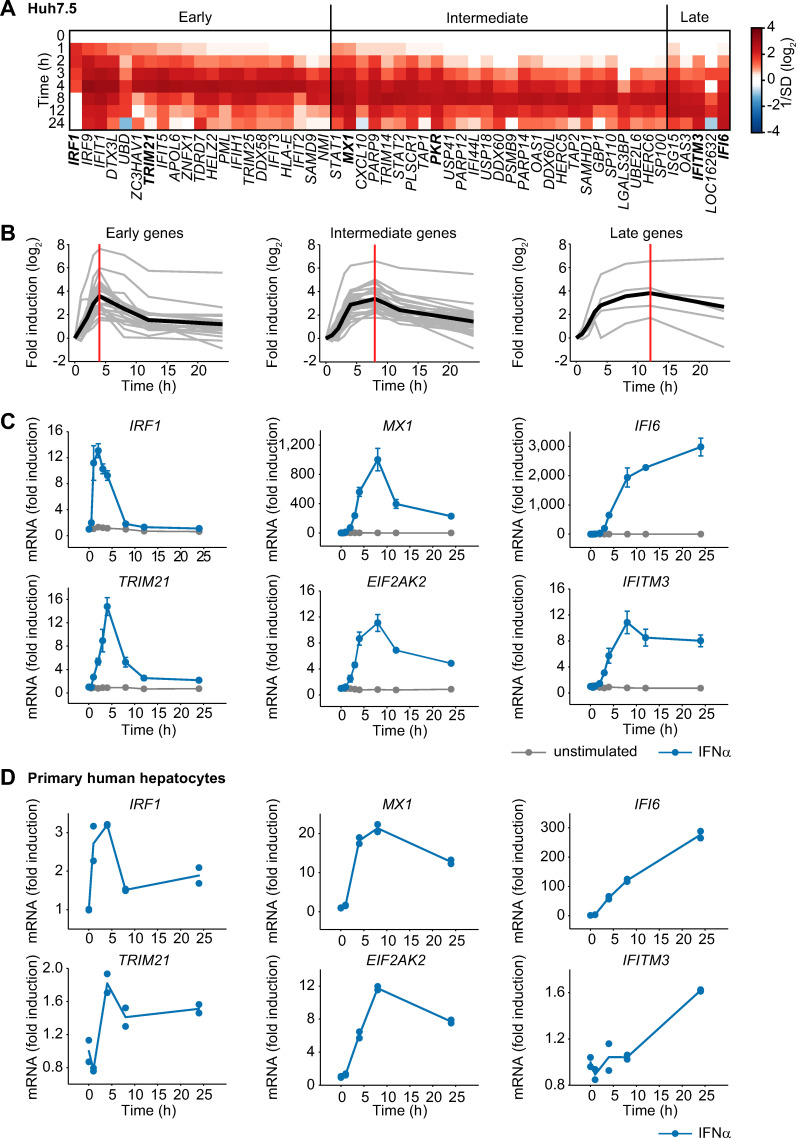
Early, intermediate and late expression profiles of IFNα-induced genes. (**A**) Microarray expression data for Huh7.5 cells stimulated with 500 U/ml IFNα. The heatmap shows the temporal expression patterns of 53 significantly upregulated genes, grouped according to their peak expression time (t_max_). Early transcripts show t_max_ ≤ 4 h, intermediate transcripts show t_max_ = 8 h and late transcripts show t_max_ ≥ 12 h. (**B**) The induction of the genes depicted in **A** is displayed in a time-resolved manner according to the respective groups (grey curves). The average expression of each group is indicated by a solid black line. The vertical red lines indicate the time points of maximal induction. (**C**) Huh7.5 cells were stimulated with 500 U/ml IFNα or left untreated and two representative antiviral genes per group were analyzed by qRT-PCR. The error bars represent SD of biological triplicates. (**D**) IFNα-induced mRNA expression in primary human hepatocytes. Primary human hepatocytes were growth factor depleted and stimulated with 500 U/ml IFNα. RNA was extracted at the indicated time points and analyzed using qRT-PCR. Points: experimental data; lines: average of duplicate measurements using primary human hepatocytes derived from one donor.

To characterize the temporal response induced by IFNα stimulation and to classify the induced genes based on their expression dynamics, we took advantage of our previously reported microarray analysis monitoring IFNα-induced gene expression over 24 hours in the human hepatoma cell line Huh7.5 stimulated with IFNα [4]. We used Huh7.5 cells as a model system, because this cell line has been widely used to investigate the replication of hepatotropic viruses. Utilizing these data, we focused our analysis on genes that exhibited significant upregulation (p<0.05 and average fold-change>2) in response to IFNα treatment (Fig 1A). Based on the time point of maximal induction (t_max_), the 53 significantly upregulated genes were classified into three expression clusters: early (t_max_ ≤ 4 h), intermediate (t_max_ = 8 h) and late (t_max_ ≥ 12 h) (Fig 1B). 21 genes classified as early were rapidly induced with a peak of maximal activation (vertical red line) one to four hours after stimulation and rapidly declined thereafter. 27 genes grouped in the intermediate cluster reached their maximal expression at around eight hours, followed by a moderate decline. Five genes were induced late and exhibited persistent upregulation with maximal expression at 12 hours or later.

As representatives for further analysis we selected two IFNα-induced genes with known antiviral acitivity from each group [20, 21]: IFN regulatory factor 1 (*IRF1*) and tripartite motif containing 21 (*TRIM21*) from the early group, MX dynamin-like GTPase 1 (*MX1*) and eukaryotic translation initiation factor 2-alpha kinase 2 (*EIF2AK2/PKR*) from the intermediate group, and IFNα-inducible protein 6 (*IFI6*) and IFN-induced transmembrane protein 3 (*IFITM3*) as examples from the late group. The characteristic dynamics of the IFNα-induced expression levels of each of the selected antiviral genes were verified by qRT-PCR analysis and confirmed the grouping into the early, intermediate and late cluster (Fig 1C).

To interrogate whether this dynamic behavior of IFNα-induced antiviral genes is characteristic for Huh7.5 cells and hence potentially determined by the cancer cell context or whether it is conserved in primary hepatocytes, we examined the IFNα-induced expression of the selected IFNα-induced antiviral genes in primary human hepatocytes isolated from a single donor. Overall the observed fold change of the expression of the IFNα-induced antiviral genes was lower in primary human hepatocytes compared to Huh7.5 cells. But in line with our previous results, the anticipated dynamic behavior was observed for each of the genes tested: The early genes *IRF1* and *TRIM21* showed maximal expression between 1 and 4 hours after IFNα treatment and rapidly declined thereafter, the intermediate genes *MX1* and *EIF2AK2* showed maximal expression between six to eight hours and rather sustained expression and the late genes *IFI6* and *IFITM3* exhibited a persistent increase for the entire observation time of up to 24 hours (Fig 1D). The conserved dynamic behavior of IFNα-induced antiviral genes in Huh7.5 cells and primary human hepatocytes suggested that the expression dynamics of IFNα-induced antiviral genes is regulated by robust mechanisms maintained in hepatocytes and possibly other cell types.

### Distinct mRNA stability affects time point of maximal induction of IFNα-induced genes

**Fig 2:**
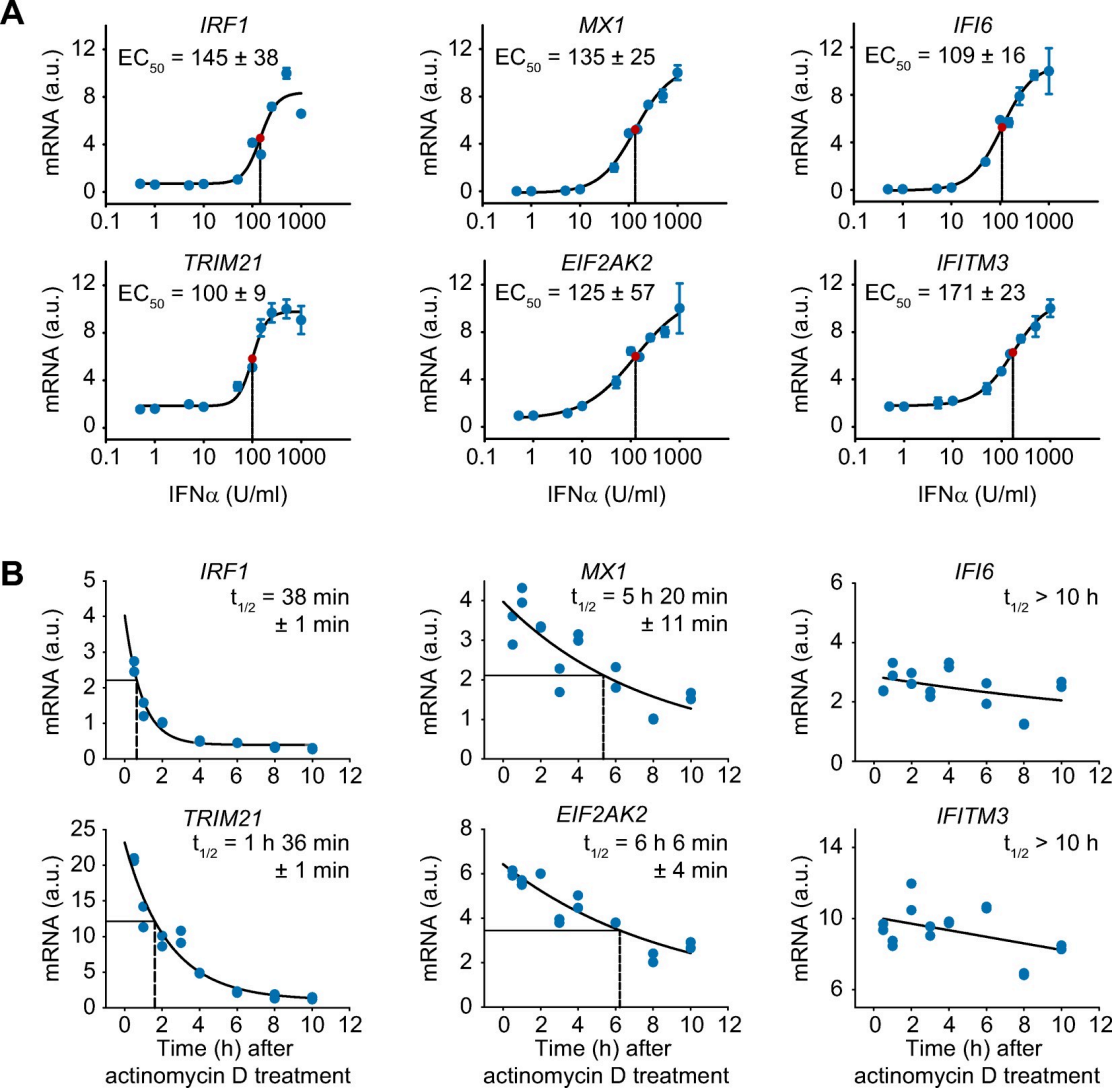
Difference in mRNA stability despite comparable dose-dependency of the expression profiles of given antiviral genes. (**A**) IFNα dose-dependent mRNA expression of antiviral genes. Huh7.5 cells were treated with increasing doses of IFNα for 4 hours. The cells were lysed, total RNA was extracted and analyzed by qRT-PCR. The error bars represent standard deviations (SD) based on biological triplicates. Regression line: sigmoidal four-parameter Hill function; red point: inflection point; dashed line: calculated EC50; a.u.: arbitrary units. (**B**) Quantification of the mRNA half-lives of the selected antiviral genes. Huh7.5 cells were stimulated with 500 U/ml IFNα for 8 hours and then treated with 5 ng/ml actinomycin D for the indicated times. Total RNA was extracted and analyzed by qRT-PCR. The data points represent biological duplicates. Regression line: three-parameter exponential decay function, dashed line: calculated RNA half-life.

To elucidate key mechanisms that contribute to the three distinct expression profiles of the IFNα-induced antiviral genes, we first tested whether the IFNα dose dependency differed between these groups. Comparing the half-maximal effective IFNα dose (EC_50_) of the selected IFNα-induced antiviral genes however showed that the EC_50_ of these genes ranged from 100 ± 9 to 171 ± 23 U/ml INFα and did not reveal substantial differences between the three groups (Fig 2A). Therefore, we next assessed whether the distinct time point of maximal induction resulted from differences in the stability of the mRNAs. To determine the mRNA half-lives of the selected IFNα-induced genes, we inhibited *de novo* transcription using actinomycin D. As shown in Fig 2B, the mRNA concentration of each of the examined antiviral genes decreased over time. To calculate the half-lives of the different mRNAs, a three-parameter exponential-decay regression was performed with the mRNA expression data. Interestingly, the time point of maximal mRNA induction of the selected genes representing the three groups were well reflected by their mRNA half-lives (Fig 2B): mRNAs that exhibited an early time point of maximal induction displayed a short half-life of 30 minutes to 2 hours; intermediate-type mRNA expression showed a half-life of approximately 5 to 7 hours; and genes with sustained-type expression profiles exhibited stable mRNAs over the entire observation period.

Thus, the three groups of IFNα-induced antiviral genes with different time points of maximal induction did not differ in their IFNα dose dependency, but were characterized by differences in mRNA stability. However, the distinct mRNA stabilities of the three groups did not explain the overall dynamics of the expression e.g. transient versus sustained expression of the antiviral genes. Therefore, we concluded that additional mechanisms such as feedback loops shape the expression profiles of IFNα-induced antiviral genes.

### Analysis of the pathway structure using a dynamic model of IFNα-induced signaling

To elucidate the potential impact of feedback loops regulating the dynamic properties of the expression of IFNα-induced antiviral genes, an ordinary differential equation (ODE) model (core model) was developed (S1A Fig). The core model was based on our previously published mathematical model [4] that was expanded by introducing mRNA expression of the negative regulators SOCS1 and USP18 and the selected IFNα-induced antiviral genes. The mathematical model was calibrated based on previously published [4] and new experimental data on the activation of the JAK/STAT pathway and IFNα-induced expression of antiviral genes that were acquired for up to 24 hours post IFNα stimulation. The initial concentrations of the main pathway components were experimentally determined (S1 Table). In addition, the experimentally determined mRNA half-life values were incorporated by introducing an mRNA-specific degradation parameter for each individual mRNA.

The simulations of the core model for the IFNα-induced signaling components (exemplarily shown for phosphorylation of JAK1 and STAT1), for the induction of the positive regulator IRF9 and for the negative regulator USP18 were consistent with the experimental data (S1B Fig). However, the trajectories of the core model were not able to reproduce the induction kinetics of the early (*IRF1* and *TRIM21*, S1C Fig) and late genes (*IFI6*, S1C Fig) as well as of the negative regulatory signaling protein SOCS1 (S1B Fig). Further, the core model failed to sufficiently reproduce the downregulation of the intermediate genes *MX1* and *EIF2AK2* indicating a missing interaction (S1C Fig). Thus, we aimed to identify missing components in our mathematical model.

### IRF2 constitutes an intracellular feedback loop that negatively regulates expression of early IFNα-induced genes

**Fig 3:**
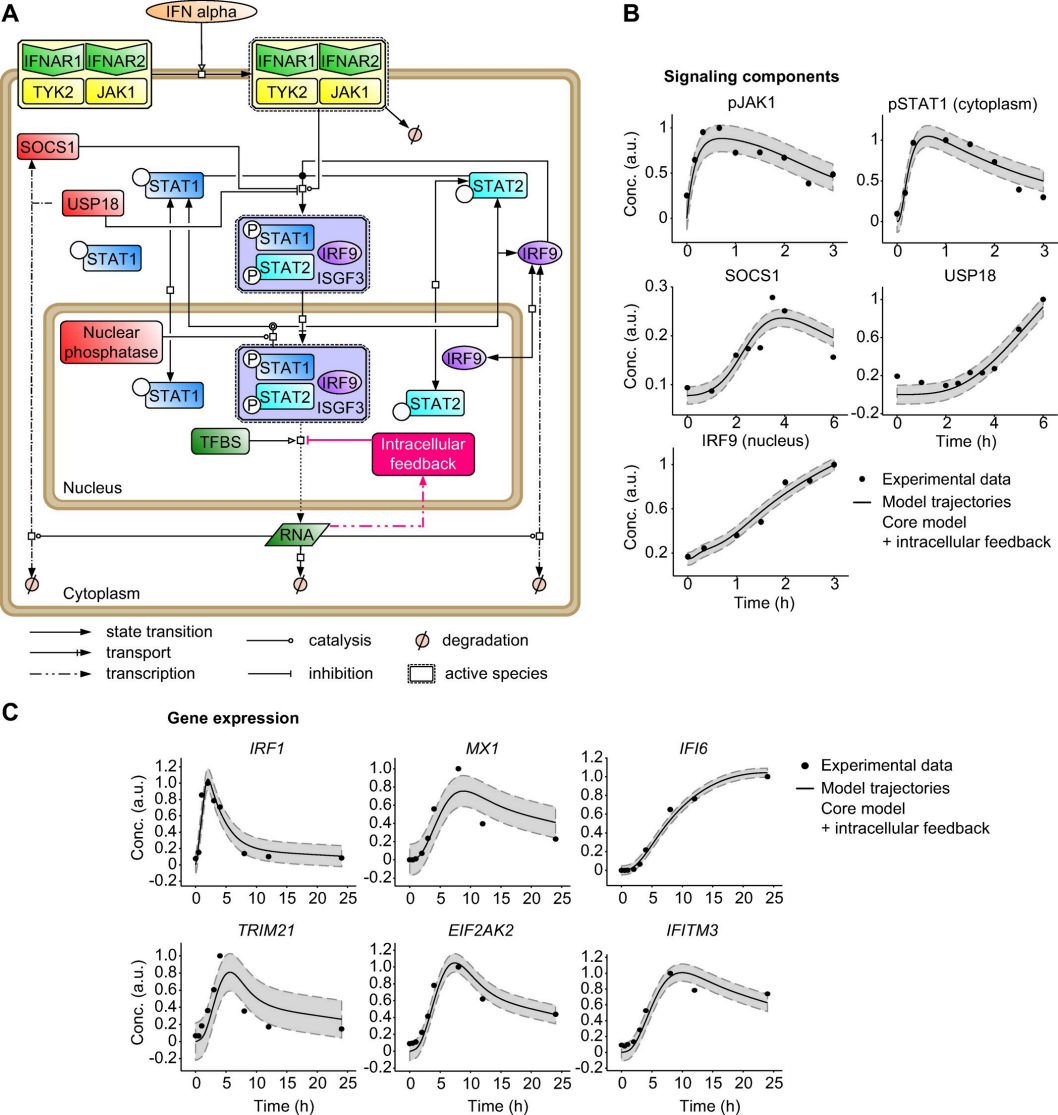
Core mathematical model with an additional intracellular feedback of IFNα-induced JAK/STAT signaling and gene expression. (**A**) Schematic representation of the core model with an additional intracellular feedback according to Systems Biology Graphical Notation. TFBS: transcription factor-binding site. (**B-C**) Trajectories of the core model with an additional intracellular feedback are shown together with the dynamic behavior of the core components of the JAK/STAT signaling pathway measured by quantitative immunoblotting (**B**) and to the expression of IFNα-induced genes examined by qRT-PCR (**C**) after stimulation of Huh7.5 cells with 500 U/ml IFNα. pJAK1 and SOCS1 were measured in cytoplasmic lysates after immunoprecipitations, pSTAT1 and USP18 were measured in cytoplasmic lysates and IRF9 was measured in nuclear lysates. Filled circles: experimental data; line: model trajectories, shades: estimated error; a.u. arbitrary units.

To improve the capacity of the model to represent the experimental data, we incorporated into the core model an additional negative feedback loop that acts exclusively at the transcriptional level (Fig 3A). As shown in Fig 3B, this model extension indeed improved the agreement between the mathematical model trajectories and the SOCS1 protein data (compare Fig 3B to S1B Fig) as well as the mRNA data for the selected IFNα-induced antiviral genes (compare Fig 3C to S1C Fig). Statistical analysis based on the likelihood ratio test (S2A Fig) and the Akaike information criterion (S2B Fig) confirmed that the core model with the additional intracellular feedback was significantly superior to the core model (S2C Fig).

**Fig 4:**
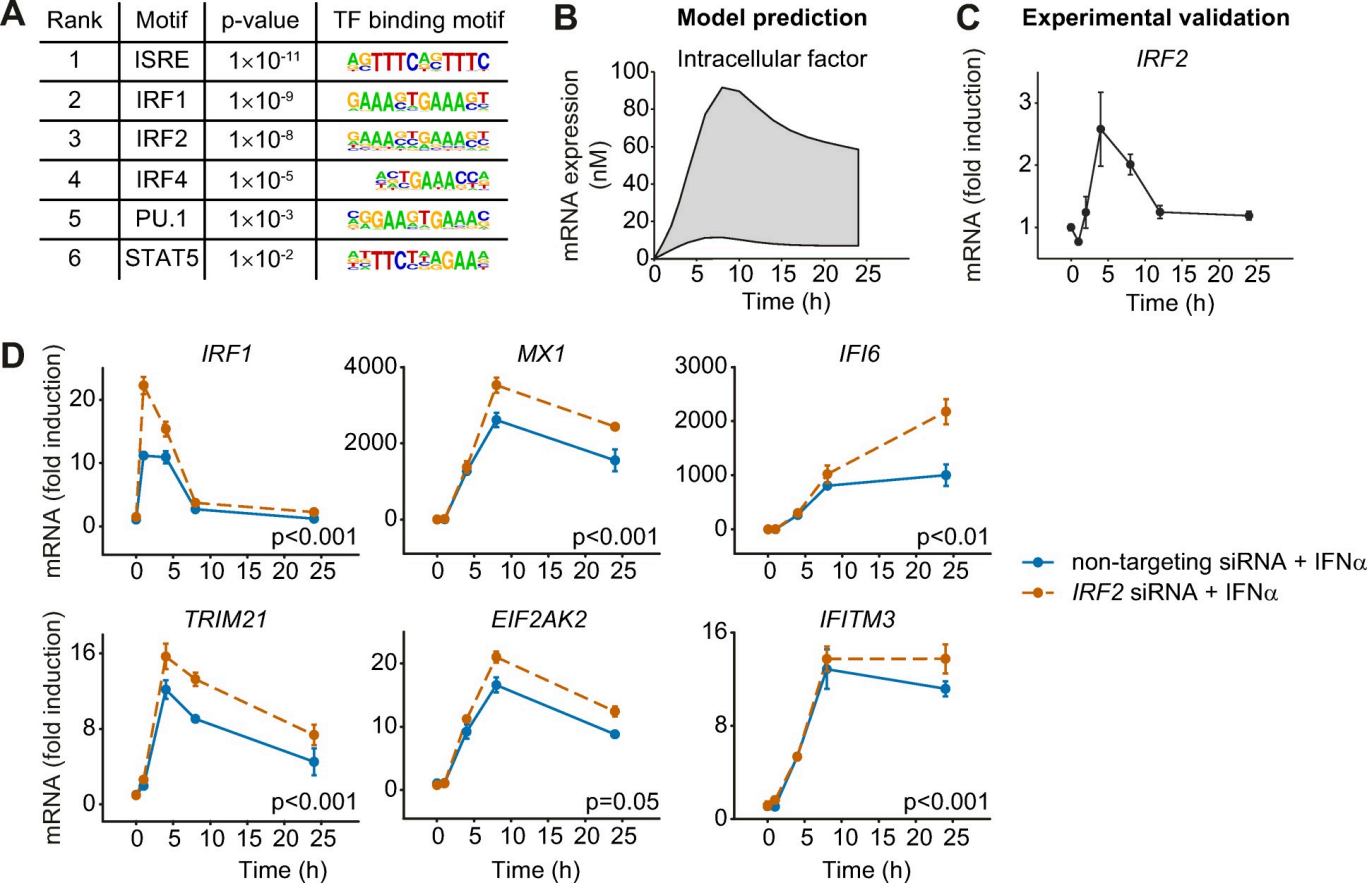
The expression profiles of the selected IFNα-stimulated genes are negatively influenced by the intracellular factor IRF2. (**A**) Transcription factor binding site analysis by the HOMER motifs software revealed six significantly regulated transcription factor binding motifs. The six most significantly enriched motifs according to the p-value and their sequence motifs are shown. (**B**) Model prediction of the mRNA expression profile of the negative regulatory intracellular factor. Shading represents the uncertainty of the prediction. (**C**) Expression profile of *IRF2* mRNA after treatment with 500 U/ml IFNα was detected by qRT-PCR. (**D**) Upregulation of gene expression by decreased *IRF2* expression. Huh7.5 cells were incubated with 50 nM siRNA directed against *IRF2* (orange) or non-targeting control (blue) for 24 hours, and then treated with 500 U/ml IFNα. The cells were lysed at the indicated time points and total RNA was extracted and analyzed by qRT-PCR. The error bars represent SD of biological triplicates. Significance was tested by 2-way ANOVA.

To identify the nature of this negative intracellular factor, we performed a transcription factor binding site (TFBS) analysis using the HOMER motive discovery approach [22]. The analysis revealed six significantly enriched transcription factor binding motifs in the genes analyzed in addition to ISRE (Fig 4A), i.e. the motifs corresponding to IRF1, IRF2, IRF4, PU.1 and STAT5. Because IRF1 is a positive regulator of antiviral genes [23], this factor was excluded. IRF2 exhibits structural similarity to IRF1 [24] but possesses a repression domain and functions as a transcriptional repressor that antagonizes IRF1-induced transcriptional activation [25]. Although IRF2 and IRF4 are structurally similar, the repressive function of IRF4 was reported to be different from that of IRF2. IRF4 possesses an autoinhibition domain of DNA binding at the carboxy-terminal region that can mask the DNA-binding domain of IRF4. PU.1, as part of the Ets-transcription factor family, forms dimers with IRF4 [26]. The presence of different proteins with similar molecular functions suggests a complex network of negative regulation of IFN-induced antiviral genes and the absence of one of these factors might be compensated by the others. To quantify the impact of the identified transcription factors, we performed siRNA knock-down experiments. Downregulation of *IRF2*, *IRF4* and *IRF8* using siRNA was performed in any possible combination. To this aim, Huh7.5 cells were incubated with siRNA directed against either *IRF2* or *IRF4* or *IRF8* or their combinations and the expression of the selected antiviral genes was monitored before and after stimulation with IFNα for 24 hours. Single downregulation with siRNA directed against *IRF2*, *IRF4* and *IRF8* showed no significant effect on the expression of the antiviral genes at the 24 hours time point. However, we observed a statistically significant upregulation of *IRF1* and *IFI6* upon treatment with combined siRNA against *IRF2* and *IRF8* compared to non-targeting siRNA (S2D Fig). These results suggest that *IRF2* or *IRF8* rather than *IRF4* affect the IFNα-induced expression of antiviral genes.

To predict the expression dynamics of the negative regulator of IFNα target gene expression, we performed simulations with our mathematical model (Fig 4B). The model predicted a slow increase with a broad peak of expression of the negative regulator of transcription around 5 to 10 hours after IFNα stimulation. The experimentally determined dynamics of *IRF4* mRNA expression revealed a minor early peak around three to four hours and a major upregulation at 24 hours after IFNα stimulation (S2E Fig). Since this expression dynamics was not in line with model-predicted expression dynamics and the siRNA results showed that knockdown of *IRF4* had no impact on the IFNα-induced target genes (S2D Fig), *IRF4* was rejected as candidate for the negative regulator of transcription. The experimentally determined dynamics of *IRF8* revealed only minor regulation upon IFNα stimulation with a downregulation after 24 hours excluding also *IRF8* as potential candidate. However, the measured expression dynamics of *IRF2* with a broad peak around five hours was similar to the dynamics predicted by the model for the expression of the negative regulator (Fig 4C). Therefore, we treated Huh7.5 cells with IFNα in combination with non-targeting siRNA or siRNA directed against *IRF2* and measured the expression profiles of the selected antiviral genes in a time-resolved manner. As shown in Fig 4D, knock-down of *IRF2* (S2G Fig) significantly enhanced the expression of all antiviral genes monitored. In line with the observed minor induction upon IFNα stimulation, knock-down of *IRF8* had no effect on the expression of the antiviral genes compared to non-targeting siRNA, while a combined knock-down of *IRF2* and *IRF8* mirrored the results obtained by siRNA against *IRF2* (S2H Fig). These results confirmed IRF2 as an important transcriptional repressor negatively regulating IFNα-induced antiviral expression and indicated that down-regulation of IRF2 leads to elevated expression of antiviral genes.

### IL1β amplifies the IFNα-induced gene response

**Fig 5:**
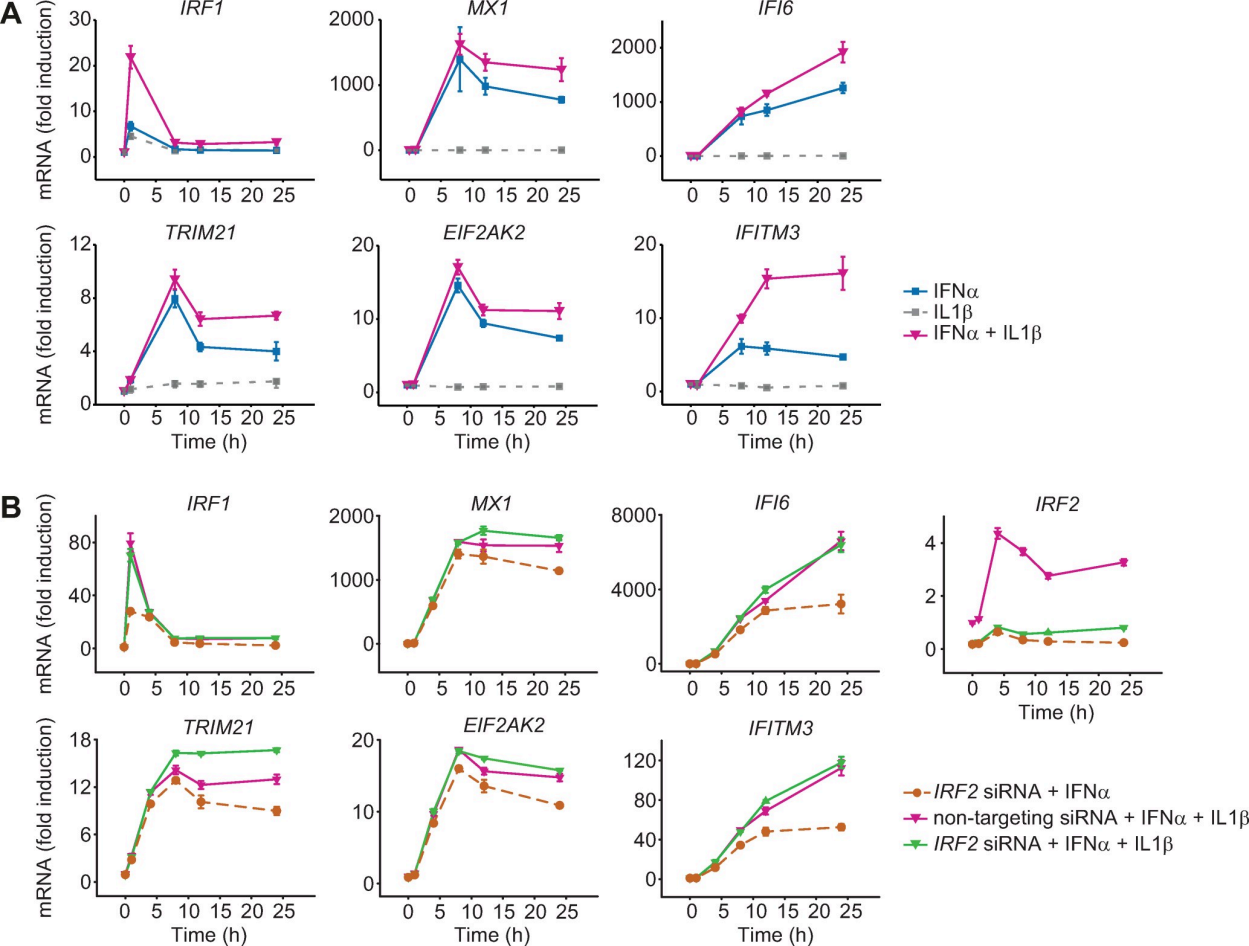
Enhanced IFNα-induced gene expression after co-stimulation with IFNα and IL1β. (**A**) IFNα-induced gene expression after co-treatment with IFNα and IL1β. Huh7.5 cells were treated with 500 U/ml IFNα, were stimulated with 10 ng/ml IL1β alone or were co-treated with 500 U/ml IFNα and 10 ng/ml IL1β. RNA was extracted at the indicated time points and analyzed by qRT-PCR. Error bars represent SD of biological triplicates. (**B**) Upregulation of gene expression by combined *IRF2* siRNA and co-treatment with IFNα and IL1β. Huh7.5 cells were incubated with 50 nM siRNA directed against *IRF2* or non-targeting control for 24 hours, and then treated with 5000 U/ml IFNα (Roferon) or co-treated with 5000 U/ml IFNα (Roferon) and 10 ng/ml IL1β. The cells were lysed at the indicated time points and total RNA was extracted and analyzed by qRT-PCR. The error bars represent SD of biological triplicates.

The observation that knock-down of a negative regulator resulted in enhanced expression of early, intermediate and late IFNα-induced antiviral genes suggested that strategies could be designed to strengthen the induction of an antiviral response. Since knock-down or inhibition of an intracellular factor is difficult to achieve *in vivo*, we tested whether an amplified expression of IFNα-induced antiviral genes could also be achieved by the addition of an extracellular factor. As it has been previously reported that cross-talk between IFNα and inflammatory cytokines may occur [27], we focused our analysis on inflammatory cytokines that are known to act in the liver: interleukin 6 (IL6), IL8 and IL1β. To experimentally test these cytokines, we performed co-stimulation experiments with each cytokine and IFNα and quantified the expression of the selected IFNα-induced antiviral genes in Huh7.5 cells. Co-stimulation with IL8 had no effect on the dynamics of IFNα-induced gene expression (S3A Fig), whereas treatment with IFNα and IL6 resulted in a small increase in the expression of the early gene *IRF1* (S3B Fig). Strikingly, co-stimulation with IFNα and IL1β resulted in markedly enhanced expression of all antiviral genes examined (Fig 5A). Stimulation of Huh7.5 cells with IL1β alone resulted only in a minor increase in the expression of *IRF1* and did not elicit the expression of the other selected antiviral genes. The enhanced expression dynamics in response to co-treatment with IFNα and IL1β resembled the effect on the expression dynamics of early, intermediate and late IFNα-induced antiviral genes observed upon knockdown of IRF2 and appeared even further elevated for the early antiviral gene *IRF1* and the late antiviral gene *IFITM3*. These results suggested that IL1β indeed can act as a strong amplifier of IFNα-induced expression of antiviral genes.

### IRF2 and IL1β independently modulate the dynamics of IFNα-induced expression of antiviral genes

To test if the IL1β-mediated enhancement of IFNα-responsive antiviral genes was dependent on IRF2, we combined knockdown of IRF2 and co-stimulation with IFNα and IL1β. We compared three conditions: IRF2 was downregulated by siRNA and Huh7.5 cells were stimulated with IFNα, a treatment that increases gene expression (compare Fig 4B). Second, we co-stimulated control cells (non-targeting siRNA) with IFNα and IL1β, another treatment that augments gene expression (compare Fig 5A). Additionally, we downregulated IRF2 by siRNA and co-stimulated the cells with IFNα and IL1β. In these three conditions we quantified the expression dynamics of the selected IFNα-induced antiviral genes and of IRF2 (Fig 5B). We observed that gene expression upon co-stimulation with IL1β and IFNα was higher in all transcripts than upon downregulation of IRF2 and stimulation with IFNα, indicating that addition of IL1β has a stronger impact than reduction of IRF2 on the IFNα-induced antiviral gene response. The combined knockdown of IRF2 and co-stimulation with IFNα and IL1β further increased gene expression compared to only co-stimulation for some of the antiviral genes such as *TRIM21*, *MX1*, *EIF2AK2*, *IFI6* and *IFITM3*, suggesting independent mechanisms of action. The dynamics of *IRF1* was not further increased by an siRNA targeting IRF2, possibly because the IFNα-induced expression of the early gene *IRF1* precedes the induction of *IRF2* expression. Interestingly, by simultaneously reducing the amount of IRF2 and co-stimulating with IL1β, genes with a transient IFNα-induced expression such as *TRIM21*, *MX1*, *EIF2AK2* exhibit a rather sustained behavior. In conclusion, we observed that the impact of IRF2 and of IL1β on the dynamics of IFNα-induced expression of antiviral genes is probably mediated by independent mechanisms and that the addition of IL1β has a stronger amplifying effect than the downmodulation of IRF2.

### IL1β-mediated STAT3 activation enhances the expression of IFNα-induced genes

**Fig 6:**
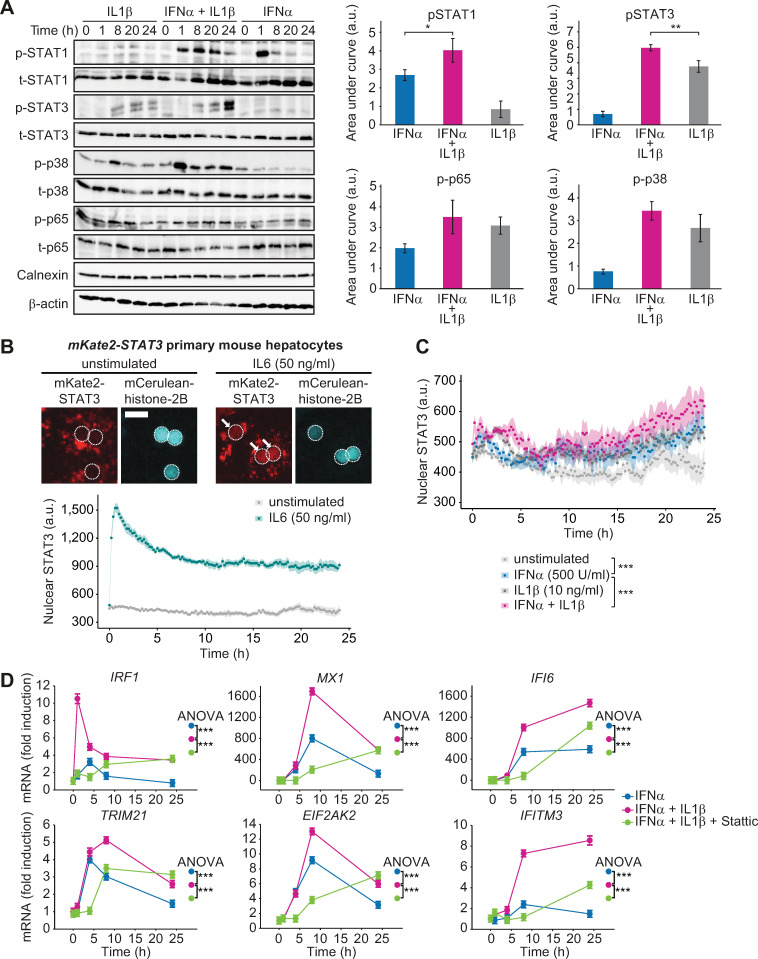
Co-stimulation of IFNα and IL1β results in phosphorylation of STAT3. (**A**) Huh7.5 cells were single or co-stimulated with 500 U/ml IFNα and 10 ng/ml IL1β. Cells were lysed at indicated time points and analyzed using quantitative immunoblotting. Error bars represent SEM of three biological replicates. (**B**) Primary mouse hepatocytes from *mKate2-Stat3* knock-in mice were growth factor depleted overnight and stimulated with 50 ng/ml IL6 or left untreated. Representative images of cells expressing a mCerulean-histone-2B nuclear marker are depicted. The dotted line indicates the outline of the nuclei and white arrows indicate nuclear STAT3. Scale bar = 25 μm. Line plots represent the dynamics of nuclear STAT3 for the indicated conditions. The median value of the nuclear mKate2-STAT3 intensity was quantified based on 5 positions per well for each replicate (four replicates derived from two mice). The mean value and SEM (N = 4) of these four median values is plotted for each time point. (**C**) Primary mouse hepatocytes from *mKate2-Stat3* knock-in mice were growth factor depleted overnight and stimulated with 500 U/ml IFNα, 10 ng/ml IL1β, co-stimulated or left untreated. The median value of the nuclear mKate2-STAT3 intensity was quantified based on 5 positions per well for each replicate (four replicates derived from two mice). The mean value and SEM (N = 4) of these four median values is plotted for each time point. Significance was tested by two-way ANOVA, ***, p<0.001. (**D**) Huh7.5 cells were pre-treated for 30 minutes with 10 μM STAT3 inhibitor Stattic followed by 500 U/ml IFNα in combination with 10 ng/ml IL1β. mRNA was extracted at indicated time points and analyzed using qRT-PCR. Error bars represent SD of biological triplicates.

It was previously reported that IL1β stimulation activates the NFκB-IκBα and the p38 signaling pathways [12]. To analyze which pathway mediated the enhancing effect of IL1β onto IFNα-induced expression of antiviral genes, Huh7.5 cells were treated with IFNα, IL1β or with a combination thereof. The dynamics of key signaling proteins in response to IFNα stimulation for up to 24 hours was analyzed by quantitative immunoblotting and for each component the area under the activation curve was calculated (Fig 6A, S3C Fig). These results showed that the phosphorylation of STAT1 was strongly induced by IFNα, but not by IL1β. However, co-treatment with IFNα and IL1β resulted in a stronger and prolonged STAT1 phosphorylation. Single IL1β treatment or co-stimulation with IFNα induced the activation of the p38 pathway and p65 of the NFκB pathway to a similar extent. Strikingly, phosphorylation of STAT3 was detected after stimulation with IL1β alone as well as after IL1β and IFNα co-treatment, whereas IFNα alone only resulted in a weak activation of STAT3. The comparison of the area under the curve of STAT3 phosphorylation showed that STAT3 phosphorylation was significantly increased in the IL1β and IFNα co-treated samples. To assess whether the increased phosphorylation of STAT3 correlated with nuclear accumulation of STAT3 in particular at late time points, we performed live cell imaging experiments with primary hepatocytes from an *mKate-Stat3* knock-in mouse strain expressing a fluorescently tagged STAT3 [28]. Compared to the treatment with IL6 that resulted in an instantaneous nuclear translocation of STAT3 (Fig 3B), nuclear STAT3 was detectable at lower levels and at later time points in response to IL1β stimulation. However, it was markedly elevated upon co-treatment with IFNα and IL1β at later time points, in particular 24 hours post treatment (Fig 6C). Therefore, the sustained STAT3 phosphorylation profiles and the nuclear accumulation of STAT3 observed upon co-treatment with IFNα and IL1β matched the co-stimulatory effect of IL1β and IFNα on the expression of the selected IFNα-induced antiviral genes.

To ascertain that STAT3 activation contributes to the enhanced expression of the selected IFNα-induced antiviral genes, single or co-stimulated Huh 7.5 cells were either left untreated or were co-treated with a STAT3 inhibitory compound (Stattic) [29]. Treatment of Huh7.5 cells with 10 μM Stattic for up to 24 hours had no significant impact on their viability (S3D Fig). With this dose of Stattic, the induction of STAT3 phosphorylation by co-stimulation with IFNα and IL1β was reduced for the entire observation time (S3E Fig). Analyzing gene expression, we noticed that at the early time points the expression of all selected genes induced by IFNα and IL1β co-stimulation was reduced by treatment with Stattic (Fig 6D). At 24 hours after IFNα and IL1β co-stimulation, expression of both early and intermediate IFNα-induced antiviral genes was comparable for Stattic-treated and untreated samples. However, the late IFNα-induced genes, *IFI6* and *IFITM3*, showed a strong decrease in their expression upon Stattic treatment during the entire observation time (Fig 6C). Overall, application of the STAT3 inhibitor Stattic had a significant effect on the expression of all analyzed IFNα-induced antiviral genes. These results indicated that co-stimulation of cells with IFNα and IL1β enhanced the activation of STAT3, thus mediating the amplified expression kinetics of IFNα-induced antiviral genes.

### IL1β enhances IFNα-induced gene expression in primary human hepatocytes and viral clearance

**Fig 7:**
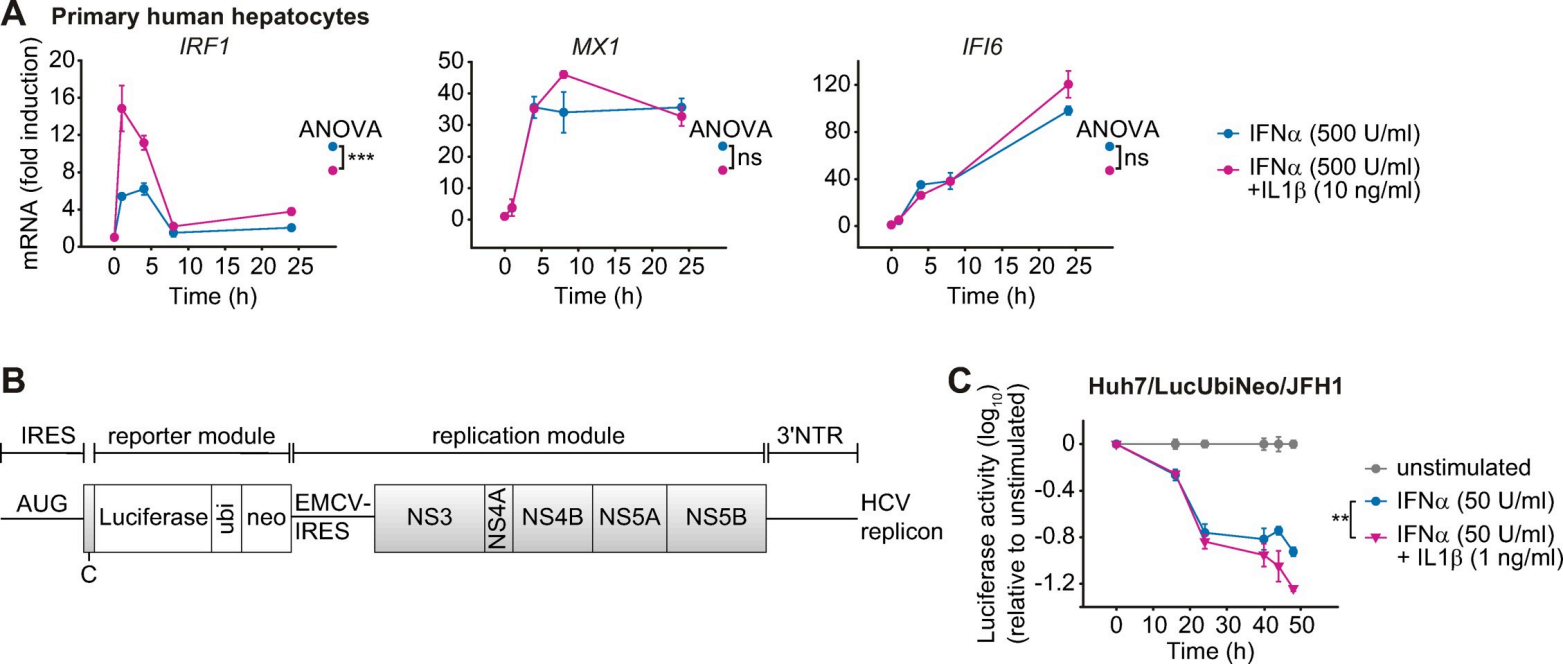
Co-stimulation with IFNα and IL1β enhances IFNα-induced gene expression in primary human hepatocytes and viral clearance of HCV in a replicon cell line. (**A**) Effect of co-stimulation with IFNα and IL1β on mRNA expression of IFNα-induced genes in primary human hepatocytes. Cells were stimulated with 500 U/ml IFNα alone or co-treated with 500 U/ml IFNα and 10 ng/ml IL1β. RNA was extracted at the indicated time points and analyzed by qRT-PCR. Triplicate samples were analyzed from three different donors and error bars represent SD. (**B**) Scheme of the bicistronic subgenomic HCV reporter RNA (replicon). IRES, internal ribosomal entry site; NTR, non-translated region. (**C**) Enhanced suppression of HCV replication in cells co-stimulated with IFNα and IL1β. Huh7/HCV/Luc replicon cells were stimulated with 50 U/ml IFNα alone or were co-treated with 50 U/ml IFNα and 1 ng/ml IL1β. The values are relative to the unstimulated control. Error bars represent the SEM of six technical replicates.

To assess whether the IL1β-induced amplification of IFNα-induced expression of antiviral genes was conserved in primary human hepatocytes and relevant for eliciting an antiviral response, we first examined the impact of IL1β on the dynamics of IFNα-induced antiviral genes in these cells. As shown in Fig 7A, consistent with our observations in Huh7.5 cells, co-stimulation of primary human hepatocytes isolated from three donors with IFNα and IL1β increased the expression especially of the early IFNα-induced gene *IRF1*. These results underscored the importance of our findings also in the context of primary human hepatocytes.

Next, we examined whether the increased expression of IFNα-induced antiviral genes in response to co-treatment with IFNα and IL1β resulted in enhanced viral clearance. For these studies we utilized a cell line containing a persistently replicating HCV reporter replicon (Huh7/LucUbiNeo/JFH1) (Fig 7B). In this cell line, luciferase activity correlates linearly with viral replication [30]. Treatment of the replicon cells with 500 U/ml IFNα–an IFNα dose that was employed in the experiment examining activation of signaling pathways or expression of antiviral genes–resulted in a very rapid inhibition of HCV replication. At this dose, a detectable but not major difference between treatment with IFNα alone and the co-stimulation with IFNα and IL1β was observed (S4A Fig). To increase the resolution of the assay and taking into account the high IFNα-sensitivity of HCV, the applied IFNα and IL1β concentrations were reduced 10-fold. In this setting, co-stimulation with IFNα and IL1β resulted in a stronger reduction in luciferase activity than IFNα alone, especially at later time points (>24 hours) (Fig 7C and S4B Fig). In conclusion, IL1β enhanced the antiviral effect of IFNα treatment and reduced HCV replication.

### IL1β-mediated enhanced expression of IFNα-induced genes requires the IL1β receptor

**Fig 8:**
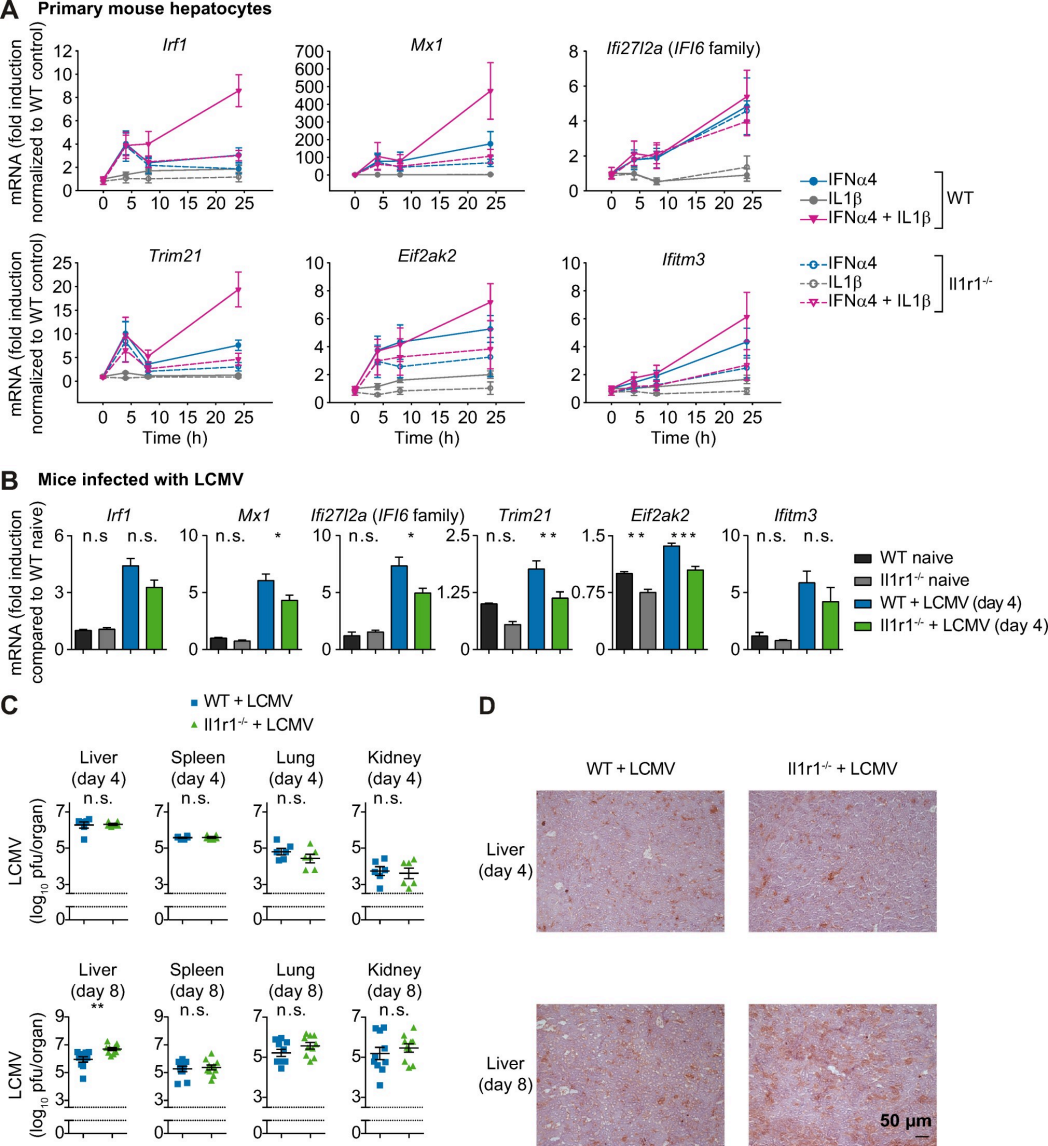
IFNα-induced antiviral response is reduced and virus replication is enhanced in Il1r1^-/-^ mice. (**A**) Expression of the selected antiviral genes in primary mouse hepatocytes from wild-type (WT) or Il1r1 knock-out (Il1r1^-/-^) mice upon stimulation with 500 U/ml murine IFNα4, 10 ng/ml murine IL1β or co-treatment. RNA was extracted at the indicated time points and analyzed by qRT-PCR. Error bars represent SEM of six (WT) and four (Il1r1^-/-^) biological replicates; a.u.: arbitrary units. (**B**) Wild-type (wt) or Il1r1 knock-out (Il1r1^-/-^) CL57BL/6 mice were infected with 2×10^6^ pfu of LCMV strain WE. Prior to and four days post infection, livers were isolated and the selected antiviral genes were measured by qRT-PCR. Differences between WT and Il1r1^-/-^ livers were tested by one-way analysis of variance. ***, p<0.001; **, p<0.01; *, p<0.05; n.s., not significant; n = 6. (**C**) Wild-type (wt) or Il1r1 knock-out (Il1r1^-/-^) CL57BL/6 mice were infected with 2∙10^6^ pfu of LCMV WE. Four and eight days post infection, livers, spleens, lungs and kidneys were isolated and viral load was quantified. Titer differences between WT and Il1r1^-/-^ organs were tested by two-sided t-tests. **, p<0.01; n.s., not significant; n = 6–10. (**D**) Wildtype (wt) or Il1r1 knock-out (Il1r1^-/-^) CL57BL/6 mice were infected with 2∙10^6^ pfu of LCMV WE. Four and eight days past infection, livers were isolated and viral proteins (LCMV-NP) were stained; n = 6–7.

To confirm the specificity of the observed augmentation of the IFNα response by IL1β, primary mouse hepatocytes were isolated from wildtype and from mice lacking the IL1 receptor (Il1r1^-/-^ mice) [31]. We measured the mouse orthologues of the human transcripts *IRF1*, *TRIM21*, *MX1*, *EIF2AK2* and *IFITM3*. Because there is no murine *Ifi6* gene, we selected *Ifi27l2a*, encoding a 7.9-kDa protein belonging to a larger family of genes comprising the *Ifi27/IFI27* genes and the human *IFI6* gene [32], because *Ifi27l2a* was identified as an interferon-stimulated gene with an antiviral role in a West Nile virus infection model [33]. To ensure that the results were not IFNα subtype-specific, we performed the experiment using two murine subtypes of IFNα, IFNα4 and IFNαA. Expression analysis of the selected murine IFNα-induced genes upon treatment with 500 U/ml murine IFNα4 or 10 ng/ml murine IL1β confirmed that treatment with IL1β alone did not induce expression of the selected IFNα-induced genes, whereas IFNα4 stimulation significantly upregulated their expression. Co-stimulation with IFNα4 and IL1β synergistically increased the expression of the selected IFNα-induced antiviral genes (Fig 8A). The experiment performed with IFNαA (S4C Fig) confirmed these results for all genes except for *Ifi27l2a*, which showed an increase in expression in the Il1r1^-/-^ samples at early time points upon IFNαA but showed no increased expression in IL1R knockout cells after stimulation with IFNα4.

IFNα-induced expression of the selected IFNα-induced antiviral genes in hepatocytes from Il1r1^-/-^ mice lacking IL1β-mediated signaling was comparable to wildtype cells for early time points. However, at later time points (24 h), IL1R knockout reduced antiviral gene expression induced by IFNα treatment alone. To analyze if in our primary hepatocyte culture not only hepatocytes were present, but also Kupffer cells that are a major source of IL1β, we stained the primary hepatocyte culture for expression of F4/80 (S4D Fig). These experiments showed that indeed a small fraction of Kupffer cells (around 4%) is present in our hepatocyte cultures, which might be responsible for the presence of low IL1β levels. Importantly, Il1r1^-/-^ cells did not show a synergistic enhancement of IFNα−induced gene expression upon co-stimulation with IL1β. These results confirmed that the co-stimulatory effect of IL1β on the IFNα-induced antiviral response is mediated by the IL1R and that the underlying mechanism is conserved in mouse and human.

### Viral replication is enhanced in Il1r1^-/-^ mice

To demonstrate the *in vivo* relevance of our findings, wildtype and Il1r1^-/-^ mice [31] were infected with 2×10^6^ pfu of LCMV stain WE. Prior to and four days post infection, the expression of the selected IFNα-induced genes in the liver of the animals was determined by qRT-PCR. In line with our hypothesis, the mRNA concentrations of the IFNα-induced genes *Mx1*, *Ifi27l2a*, *Trim21* and *Eif2ak2* were significantly reduced in infected Il1r1^-/-^ mice compared to wildtype mice (Fig 8B). We measured viral titers in four different tissues (liver, spleen, lung and kidney) four days and eight days post infection. As comparable virus amounts were detected four days post infection (Fig 8C) in the liver, the reduction in antiviral genes detected in Fig 8B was not due to differences in viral load. However, in line with the reduced antiviral response in the liver, we observed a significant increase in LCMV titers in the liver of Il1r1^-/-^ mice as compared to wildtype controls eight days post infection in the liver (Fig 8C). Consistently, immunohistochemical evaluation of liver tissue revealed that LCMV nucleoprotein (NP) was more abundant in hepatocytes of Il1r1 deficient mice than in wildtype counterparts (Fig 8D). During LCMV infection, type I IFNs can limit viral replication [34], but also protect anti-viral T cells from NK cell-mediated attack [35, 36]. T cell immunity is critical for LCMV control, and viral persistence can result in exhaustion of T cells [37]. Hence, we monitored anti-viral T cells in IL1R knockout mice following infection. Notably, antiviral T-cell immunity was reduced eight days post infection following LCMV infection in Il1r1^-/-^ compared to control animals (S5A–S5C Fig). In conclusion, the *in vivo* experiments confirmed the importance of the IL1β induced signal transduction mediated by the IL1 receptor for enhancing the IFNα-induced response.

## Discussion

We observed that the temporal expression profiles of IFNα-induced genes can be classified into three different groups based on the time point of maximal activation: early, intermediate and late. By mathematical modeling based on time-resolved experimental data, our studies revealed that mRNA stability and expression of IRF2 as a negative regulator of transcription critically determine the expression profiles of IFNα-induced genes. Strikingly, we observed that IL1β-induced enhancement of gene expression functionally resembles the impact of IRF2 knockdown and significantly boosts IFNα-induced responses. Because IRF2 impacts gene expression as a transcriptional repressor, while IL1β induces enhanced gene expression via STAT3 activation, we here describe two distinct regulatory modes that converge at the promoter level of the IFNα-induced antiviral genes. Functional mimicry can be achieved by downregulation of IRF2 or addition of IL1β, both resulting in enhanced IFNα-induced gene expression, with IL1β having the dominant effect. Combined knockdown of IRF2 and co-stimulation with IL1β even further increased the IFNα-induced gene expression.

It has previously been reported that TNF stimulation of mouse fibroblasts for twelve hours resulted in early, intermediate and late gene expression clusters and that these clusters differ in mRNA stability [38]. Consistent with these observations, we demonstrated that the mRNA half-lives of the IFNα-induced antiviral genes indeed differ substantially among the three groups and correlate with their peak of expression. In line with these results, an analysis of publicly available data sets, including data sets from LPS-treated macrophages and IFNγ-stimulated bone marrow macrophages, revealed a highly significant correlation between the observed mRNA peak time and the corresponding mRNA stability in most experiments [39]. Furthermore, it was shown that pro-inflammatory stimuli increase the transcription rate of mRNAs in mouse dendritic cells, while the response duration is mainly determined by the RNA decay rates [40]. We therefore conclude that mRNA stability might be a general cellular mechanism controlling the peak time of innate immune gene expression. In our study, the clustering of transcripts into early, immediate and late correlated with the corresponding mRNA stability, but mRNA stability alone was not sufficient to explain the mRNA dynamics upon IFNα stimulation.

Positive and negative feedback mechanisms establish a balanced regulatory network of type I IFN-induced signaling [1] and the combination of transcriptional activators and repressors is critical for the expression of specific genes and viral clearance. Consistent with previous results [25], we observed that the transcription factor IRF2 is induced by IFNα. In addition, we demonstrated that the downregulation of IRF2 by siRNA enhances antiviral gene expression, which is in agreement with an elevated IFN-induced gene expression in IRF2-deficient mice [41]. Furthermore, it has been demonstrated that IRF2 knock-down results in the upregulation of IFN-induced genes in the bone marrow [42]. Virus-induced IFNβ expression is substantially higher in IRF2-deficient mice than in wild-type mice [43], and HCV-infected patients exhibit increased expression of IRF2 [44]. In line with these observations, we showed that IRF2 negatively regulates the expression of IFNα-induced genes and represents an important feedback mechanism dampening the type I IFN response.

Our experimental studies and the analysis by the mathematical model showed that mRNA stability is intrinsic to the antiviral genes examined and neither affected by IFNα nor IRF2. Rather, IRF2 negatively affects the expression of these IFNα-induced transcripts via the IRF2 transcription factor binding site. The core mathematical model, which only takes differences in mRNA stability of individual ISGs into account, was unable to describe the IFNα-induced gene expression dynamics. On the contrary, the mathematical model that considers the presence of an additional negative transcriptional regulator is capable to describe the observed IFNα-induced expression dynamics of the antiviral genes. Our studies identified IRF2 as this negative regulator dampening ISG expression.

Additionally, we provided evidence that co-stimulation with IL1β enhances the expression of IFNα-induced genes. In agreement with this observation, it was previously reported that IFNα and IL1β co-stimulation in Huh7.5 cells increased the phosphorylation of STAT1 and resulted in an increased expression of two antiviral proteins, PKR (encoded by *EIF2AK2*) and OAS, compared to treatment with IFNα alone [19]. Furthermore, it was reported in HepG2 cells that prestimulation with IL1β attenuates IFNα-induced STAT1 activation by a proteasome- and NFκB-dependent mechanism [45]. In our study, co-stimulation with IFNα and IL1β rather shifted the peak of STAT1 phosphorylation to later time points and we could not find evidence of involvement of the NFκB pathway (Fig 6A and S3C Fig).

We further showed that IL1β stimulation strikingly induced the phosphorylation of STAT3 at time points later than 6 hours. The IL1β-induced activation profile of STAT3 was remarkably different from the IL6-induced STAT3 phosphorylation that peaks at one hour after stimulation. At present, there are only very few reports on STAT3 activation by IL1β. For example, IL1β-induced phosphorylation of STAT3 was reported in myocytes [46], in mesangial cells [47] and in HepG2 cells with a weak increase of phosphorylation eight hours after stimulation [13]. In our study, inhibition of STAT3 activation by the treatment with the inhibitor Stattic reduced the IL1β-induced STAT3 phosporylation and the expression of antiviral genes after IFNα and IL1β co-stimulation. Likewise, in RAW 264.7 cells, a reduction of LPS-induced STAT3 activation and target gene expression was observed upon treatment with the inhibitor Stattic [48]. In conclusion, this is to our knowledge the first report indicating that IL1β stimulation triggers prolonged STAT3 phosphorylation and nuclear translocation.

We thus observed that on the one hand IRF2 negatively regulates IFNα-induced expression of antiviral genes, most likely via the transcription factor binding site detected by our bioinformatics approach. On the other hand, co-stimulation with IL1β enhanced IFNα-induced expression of antiviral genes. In line with this result, Huh7 cells transfected with STAT3 shRNA were previously reported to show reduced induction of several IFNα-induced target genes, including *EIF2AK2* (PKR) [49]. Because IL1β stimulation alone did not result in a significant upregulation of the antiviral genes analyzed, neither in Huh7.5 cells (Fig 5A) nor in primary mouse hepatocytes (Fig 8A), we concluded that both IFNα-induced STAT1/STAT2 and IL1β-induced STAT3 have to be simultaneously activated for an enhanced antiviral response. A possible molecular link between phosphorylated STAT3 and the IFNα target genes could be via complexes of the transcription factors. Previously, interactions of STAT1 with STAT3 were detected in IL6-stimulated HepG2 cells [50] and complexes of STAT2 with STAT3 were shown in IFNα-stimulated U266 cells [51]. Of note, co-stimulation with IL6, a strong inducer of STAT3 nuclear translocation (Fig 6B), was only inducing a minor enhancement in IFNα-induced expression of *IRF1*, but not of *MX1* and *IFIF6* (S3B Fig). It was previously reported that, e.g. in myeloid cells, complex formation of STAT3 with STAT1 can result in sequestration of STAT1 [52]. In a previous study, we have shown that IFNα induces the formation of the different STAT1 containing complexes in a temporal and dose-dependent order based on the number of STAT1, STAT2 and IRF9 molecules per cell [10], indicating that the abundance and the dynamics of activation of the complex components critically determine which transcription factor is formed. We therefore speculate that for the enhanced induction of antiviral genes observed upon co-stimulation with IL1β and IFNα, a low but sustained activation of STAT3 is required to favor complex formation with STAT1 and to allow DNA binding of the resulting complex. A strong activation, as elicited by IL6, in contrast leads to sequestration of STAT1 and thereby prevents DNA binding of STAT1-containing complexes.

In Huh7.5 cells, IL1β on its own did not cause an upregulation of the IFNα-induced antiviral genes in the observed time frame of 24 h, whereas co-treatment of IL1β with IFNα elevated their expression compared to IFNα stimulation alone. However, in cultured primary mouse hepatocytes, the knockout of the IL1β receptor reduced the antiviral gene expression triggered by IFNα treatment alone at the 24 h time point. We speculate that the observed effect is due to residual IL1β secreted by a small fraction of Kupffer cells that is present in the cultures of primary hepatocytes, which was confirmed by staining with antibodies recognizing F4/80 [53]. Although hepatocytes constitute the major cell type in the liver, a significant fraction of non-parenchymal cells such as Kupffer cells, sinusoidal endothelial cells and hepatic stellate cells are also present and isolation of hepatocytes at 100% purity is currently not possible [54]. Furthermore, even very low and barely detectable levels of IL1β are capable to induce robust signal transduction in hepatocytes, which are more sensitive to low concentrations of IL1β than other cell types such as macrophages [55].

While our experimental system was specifically geared towards IFNα, the receptor and the activated signal transduction cascade is identical for all type I IFNs, including the very early IFNβ [56]. Hence, we assume that the enhancing effect of IL1β also affects IFNβ signaling. Of note, however, IFNβ represents an immediate early response of infected cells [57], and plays a major role during the initial phase of infection, likely before IL1β-producing cells are recruited to and become activated at the site of infection. In contrast, later stages of infection are likely to be affected by the synergy between IFNα and IL1β, as indicated by the enhanced antiviral state inferred from the increased inhibition of LCMV replication in the mouse model.

In this *in vivo* setting we furthermore demonstrated that hepatic viral titers were significantly increased in IL1R knockout mice, while significantly reduced expression of IFN stimulated genes were observed in the liver. In addition, our data indicated that IL1R knockout mice show reduced anti-viral T cells in the spleen following infection, but no increase in LCMV titers in the spleen or other organs were observed. Therefore, we speculate that in the liver local type I IFN responses are boosted by the presence of IL1β likely secreted by non-parenchymal cells. However, for final confirmation future studies with liver-specific IL1β receptor knockout mice would be required [58]. Clinical data demonstrated that levels of pro-inflammatory cytokines including IL1β, IL4 and IL6 are elevated in the sera of patients with HCV infection [59]. However, the role of IL1β in hepatitis virus-infected individuals and the impact on viral clearance are controversially discussed. On the one hand, it was reported that IL1β concentrations are within the normal range during IFNα treatment of HCV patients [60] and decrease in chronically infected patients [61]. On the other hand, Daniels *et al*. demonstrated that the increased production of IL1β by peripheral blood mononuclear cells during IFNα treatment contributes to the inhibition of hepatitis B virus replication and promotes viral clearance [62]. Similarly, Zhu *et al*. reported that IL1β inhibits HCV replication in a hepatoma-derived replicon cell line [63].

In conclusion, we demonstrate that IL1β boosts the expression of IFNα-induced antiviral genes, and in vivo particularly those with an intermediate and a late expression profile. IL1β thereby could strengthen the efficacy of therapeutically applied IFNα in particular in the liver and this knowledge might help to improve IFN-based strategies for the treatment of viral infections.

## Materials and methods

### Cell culture

Huh7.5 cells were kindly provided by Charles M. Rice (The Rockefeller University, NY, RRID:CVCL_7927) and primary human hepatocytes (PHH) were kindly provided by Georg Damm (Charité Berlin). Murine hepatocytes were isolated from wildtype or from Il1r1^-/-^ CL57BL/6 mice as previously described [64].

The SOP for cultivation of Huh7.5 cells are available from protocols.io (dx.doi.org/10.17504/protocols.io.biapkadn). All cells were cultivated at 37°C and 5% CO_2_ incubation and 95% relative humidity. Informed consent of the patients for the use of tissue for research purposes was obtained corresponding to the ethical guidelines of the Charité-Universitätsmedizin Berlin. The Huh7.5 cell line was authenticated using Multiplex Cell Authentication and the purity of cell line was validated using the Multiplex Cell Contamination Test by Multiplexion (Heidelberg, Germany) as described recently [65, 66].

### Cells stimulation for protein and mRNA measurements

One day before time-course experiments, 1.7∙10^6^ Huh7.5 cells or 2∙10^6^ PHH were seeded into 6 cm-diameter dishes or 5.5∙10^5^ cells per well of 6-well plates in culture medium. Huh7.5 were cultured in Dulbeccos’s Modified Eagle Medium (DMEM, Invitrogen) supplemented with 1% Glutamax, 10% fetal calf serum (FCS) (Gibco) and 1% P/S (Invitrogen). PHHs were cultivated in Williams medium E (Biochrom) supplemented with 10% FCS (Gibco), 100 nM dexamethasone, 10 μg/ml insulin, 2 mM L-Glutamin (Gibco) and 1% Penicillin-Streptomycin (P/S) (Invitrogen). Prior to stimulation, cells were washed three times with PBS and cultivated in serum-free medium for three hours. Stimulation of cells was performed by adding the stimulation factor directly into serum free medium. To stop stimulation, dishes were placed on ice, medium was aspirated and cells were lysed either with Nonidet P-40 lysis buffer (1% NP40, 150 mM NaCl, 20 mM Tris pH 7.4, 10 mM NaF, 1 mM EDTA pH 8.0, 1 mM ZnCl_2_ pH 4, 1 mM MgCl_2_, 1 mM Na_3_VO_4_, 10% Glycerol and freshly added 2 μg/ml aprotinin and 200 μg/ml AEBSF) or Nonidet P-40 cytoplasmic lysis buffer (0.4% NP40, 10 mM HEPES pH 7.9, 10 mM KCl, 0.1 mM EDTA, 0.1 mM EGTA and freshly added 2 μg/ml aprotinin, 200 μg/ml AEBSF, 1 mM DTT, 1 mM NaF and 0.1 mM Na_3_VO_4_) and nuclear lysis buffer (20 mM HEPES pH 7.9, 25% glycerin, 400 mM NaCl, 1 mM EDTA, 1 mM EGTA and freshly added 2 μg/ml aprotinin, 200 μg/ml AEBSF, 1 mM DTT, 1 mM NaF and 0.1 mM Na_3_VO_4_) for cell fractionation. To measure the viability of cells upon Stattic treatment, CellTiter-Blue Viability Assays (Promega) were performed according to the manufacturer’s instructions. Incubation with the dye for 60 min was followed by measurement of the fluorescence with the infinite F200 pro Reader (Tecan).

### RNA analysis

Cells were seeded, growth factor depleted and stimulated with IFNα (PBL, 11350–1). For Fig 5B, Roferon (Roche) was used. Because more Roferon is required to obtain equipotent doses to research grade IFNα [10], 5000 U/ml of Roferon was used in this experiment. Total RNA was isolated from three independent dishes per time point by passing the lysate through a QIAshredder (Qiagen) for homogenization, followed by RNA extraction using the RNeasy Plus Mini Kit (Qiagen) according to manufacturer’s protocol. For cDNA generation, 1 μg of total RNA was used and transcribed with the High-Capacity cDNA Reverse Transcription Kit (Applied Biosystems) according to manufacturer’s instructions. Quantitative real-time PCR (qRT-PCR) was performed using the hydrolysis-based Universal Probe Library (UPL) platform (Roche Diagnostics) in combination with the Light Cycler 480 (Roche Diagnostics). Primers were generated using the automated Assay Design Center based on species and accession number (www.lifescience.roche.com) (see S2 Table). Crossing point (CP) values were calculated using the second derivative maximum method of the Light Cycler 480 software (Roche Diagnostics). An internal dilution series of template cDNA (stimulated for 1 hour with 500 U/ml IFNα) was measured with every gene analyzed for PCR efficiency correction and served as standard curve for calculation of relative concentrations. Relative concentrations were normalized to *HPRT*.

### Quantitative immunoblotting

For immunoprecipitation (IP), the target-specific antibody was added to the cellular lysates together with 25 μl of Protein A or G sepharose (GE Healthcare) depending on the species of target antibody and the mixture was incubated overnight rotating at 4°C. For anti-JAK1 (Upstate Millipore, 06–272, RRID:AB_310087), anti-Tyk2 (Upstate Millipore, 06–638, RRID:AB_310197) and anti-STAT1 (Upstate Millipore, 06–501, RRID:AB_310145) IP, Protein A sepharose was used. Protein G sepharose was used for anti-SOCS1 (Millipore, 04–002, RRID:AB_612104) IP. Protein concentration of cellular lysates was determined using the BCA Assay kit (Pierce/Thermo Scientific) according to the manufacturer’s instructions. Proteins were separated by denaturing 10% or 15% SDS-PAGE. Sample loading was randomized to avoid systematic errors [67]. The proteins were transferred to PVDF (STATs, IRF9, USP18) or nitrocellulose membranes (JAK1, TYK2). Membranes were stained with 0.1% Ponceau Red (Sigma-Aldrich). To detect tyrosine phosphorylation of immunoprecipitated JAK1 and TYK2, the anti-phosphotyrosine monoclonal antibody 4G10 (Upstate Biotechnology, 05–321, RRID:AB_309678) was used. A list of antibodies employed in this study is shown in Table 1.

**Table 1:** Antibodies employed in this study.

Antibody	Vendor, CatLog	RRID
anti-phospho-STAT1	Cell Signaling Technologies, 9171	RRID:AB_331591
anti-phospho-STAT2	Cell Signaling Technologies, 4441	RRID:AB_2198445
anti-IRF9	BD Transduction Laboratories, 610285	RRID:AB_397680
anti-USP18	Cell Signaling Technologies, 4813	RRID:AB_10614342
anti-SOCS1	Invitrogen, 04–002	RRID:AB_612104
anti-phospho-p38	Cell Signaling Technologies, 4511	RRID:AB_2139682
anti-phospho-p65	Cell Signaling Technologies, 3031	RRID:AB_330559
anti-JAK1	Cell Signaling Technologies, 06–272	RRID:AB_310087
anti-TYK2	Upstate Millipore, 06–638	RRID:AB_310197
anti-STAT1	Upstate Millipore, 06–501	RRID:AB_310145
anti-STAT2	Upstate Millipore, 06–502	RRID:AB_310146
anti-p38	Cell Signaling Technologies, 9212	RRID:AB_330713
anti-p65	Santa Cruz, sc-109	RRID:AB_632039
anti-calnexin	Enzo life sciences	RRID:AB_10616095
anti-β-actin	Sigma Aldrich, A5441	RRID:AB_476744
anti-PARP	Roche	RRID:AB_1602926

For detection of additional proteins on the same membrane, membranes were incubated with β-mercaptoethanol and SDS. For normalization, antibodies against calnexin and β-actin were used for the cytoplasmic fraction and anti-PARP was used for the nuclear fraction. Secondary horseradish peroxidase-coupled antibodies (anti-rabbit HRP, anti-goat HRP, Protein A HRP) were purchased from GE Healthcare. Immunoblots were incubated with ECL or ECL advance substrate (GE Healthcare) and signals were detected with a CCD camera (ImageQuant LAS 4000 biomolecular imager (GE Healthcare)). Immunoblot data was quantified using ImageQuant TL version 7.0 software (GE Healthcare). Quantitative immunoblot data were processed using GelInspector software [67]. Data normalization was performed by using either the recombinant calibrator proteins GST-JAK1ΔN or GST-Tyk2ΔC for JAK1 and TYK2, respectively, or housekeeping proteins: β-actin for IRF1, IRF9, USP18, p38 and p65 or calnexin and PARP for STAT1 and STAT2 in the cytoplasm and nucleus, respectively. For smoothing splines to the data, Matlab’s csaps-splines with a smoothing parameter of 0.8 were used.

### siRNA transfection

For siRNA transfection, 2.25×10^**5**^ Huh7.5 cells were seeded in 6-well plates 24 hours prior to transfection. The next day, cells were washed three times with PBS and cultivated in P/S free DMEM supplemented with 10% FCS before the transfection with 50 nM siRNA (Dharmacon) (*IRF2*: L-011705-02-0005; *IRF4*: L-019668-00-0005; *IRF8*: L-011699-00-0005; non-targeting siRNA: D-001810-10-20). Transfection was performed by incubation of siRNA with Optimem Medium (Gibco, Life Technologies) and Lipofectamin RNAiMAX (Invitrogen) for 20 minutes at RT and adding the mixture dropwise to cells. For efficient uptake, cells were incubated with siRNA transfection mixture for 24 hours. Subsequently, the medium was changed and time course experiments were performed.

### Live-cell imaging

Primary hepatocytes (15,000 cells per well, 96-well plate format) derived from *mKate2-STAT3* heterozygous knock-in mice [28] were transduced with adeno-associated viruses encoding mCerulean-labeled histone-2B during adhesion. Cells were cultivated as described above, stimulated with ligand, and imaged using a Nikon Eclipse Ti Fluorescence microscope in combination with NIS-Elements software. Temperature (37°C), CO_2_ (5%) and humidity were held constant through an incubation chamber enclosing the microscope. Three channels were acquired for each position: bright-field channel, STAT3 channel (mKate2), and nuclear channel (CFP). Image analysis was performed using Fiji software and data were processed using R software. Briefly, the median value of the nuclear mKate2-STAT3 intensity was quantified based on 5 positions per well for each replicate (four replicates derived from two mice). Afterwards, the mean value and SEM (N = 4) of these four median values were calculated for each time point.

### Luciferase assay

Luciferase activity was measured as read out for HCV replication. 30,000 cells of the replicon cell line Huh7/LucUbiNeo/JFH1 [68] were seeded in a 24-well plate two days prior to the stimulation. Cells were growth factor depleted for 3 hours followed by IFNα treatment. At different time points cells were washed once with PBS and lysed with 100 μl luciferase lysis buffer (1% Triton X-100, 25 mM glycil-glycin (pH 7.8), 15 mM MgSO_4_, 4 mM EGTA, 10% Gylcerol) directly in the well. Plates were stored at -80°C until measurement. Luciferase was measured applying 400 μl luciferase assay buffer (15 mM K_3_PO_4_ (pH7.8), 25 mM glycil-glycin (pH 7.8), 15 mM MgSO_4_, 4 mM EGTA) with freshly added 1 mM DTT, 2 mM ATP and 1mM D-Luciferin. Luciferase activity was measured using Mitras^2^ multimode reader LB942 (Berthold).

### Cultivation of primary mouse hepatocytes

Cells were seeded with a density of 3.5×10^5^ cells per cavity in a collagen-coated 6-well-plate. For the experiments cells were cultivated under FCS-free conditions in DMEM/Ham’s F-12 (Biochrom) supplemented with 2 mM glutamine and 100 U/ml penicillin/0.1 mg/ml streptomycin (Cytogen). Cells were stimulated with 500 U/ml of murine recombinant IFNα4 (Fig 8A) or IFNαA (S4C Fig) (PBL) with or without 10 ng/ml of murine recombinant IL1β (JenaBioscience) for the time points indicated in the respective figure.

### Immunofluorescent staining and microscopy

Isolated murine hepatocytes from three animals were seeded on glass coverslips and fixed with methanol at -20°C for 5 minutes. Thereafter, samples were washed four times at room temperature with phosphate-buffered saline (PBS) w/o Ca^2+^ or Mg^2+^ and then blocked with 5% bovine serum albumin (BSA) for one hour. Next, coverslips were incubated with primary antibodies diluted 1:200 in blocking buffer targeting F4/80 (rat anti-mouse from AbD Serotec/Bio-Rad, Cat. No. MCA-497RT) for 90 minutes at room temperature. After additional washing four times with PBS, coverslips were incubated with fluorochrome-conjugated secondary antibodies diluted 1:200 in blocking buffer (FITC-labeled donkey-anti-rat from Dako/Agilent, Cat. No. 712-485-150) and Hoechst 33258 staining solution diluted 1:20,000 (Sigma-Aldrich). Following a four times washing step with PBS, coverslips were covered with mounting medium (Dako/Agilent) and placed on object slides. Stained samples were analyzed by microscopy using the cell observer system from Zeiss (Jena, Germany). 8 to 10 color images per animal (including primary antibody) and 4 images (2nd antibody control) were recorded with a 25-fold magnification. The total number of cells for each image (368 cells in average) was counted based on the Hoechst signal and the number of F4/80-positive cells was determined for each image. In total, 31 images were analyzed. The ratio of F4/80-positive cells to the total number of cells was calculated for each image and the results were displayed as box plots.

### RNA isolation and qRT-PCR of primary mouse hepatocytes

Total cellular RNA was isolated by using the RNeasy Miniprep Kit (Qiagen) as described in the manufacturer’s instructions. 1 μg of total RNA was reverse transcribed with Quantitect Reverse Transcription Kit (Qiagen) using oligo(dT), which included DNase I digestion. cDNA was diluted 1/5, and 1.2 μl of the diluted cDNA was added as template to a final volume of 25 μl including 1x GoTaq qPCR Master Mix according to the manufacturer’s instructions (Promega, Mannheim, Germany). qRT-PCR was performed using the ViiA7 real-time PCR system (Applied Biosystems). Primers were generated using the Primer-BLAST design tool from NCBI based on the accession number of the gene of interest. All primers were purchased from Eurofins MWG Operon (Ebersberg). Specificity of rtPCR was controlled by no template and no reverse-transcriptase controls. Semiquantitative PCR results were obtained using the ΔCT method. As control gene *Hprt* was used. Threshold values were normalized to *Hprt* respectively.

RNA purification of liver tissue from LCMV infected mice for qRT-PCR analyses were performed as previously described [69]. Gene expression of *Irf1*, *Mx1*, *Isg12*, *Trim21*, *Eif2ak2*, *Ifitm3*, *Hprt* was performed using kits from Applied Biosystems. For analysis, the expression levels of all target genes were normalized to *Hprt* expression (ΔCt). Gene expression values were then calculated based on the ΔΔCt method, using the naïve liver samples as a control to which all other samples were compared. Relative quantities (RQ) were determined using the equation: RQ = 2^-ΔΔCt^.

### LCMV infection of wild-type or Il1r1 knock-out mice

All mice were on a C57BL/6 genetic background. Il1r1^-/-^ mice [31] were obtained from Jackson Laboratory (mouse strain 003245). LCMV strain WE was originally obtained from F. Lehmann-Grube (Heinrich Pette Institute, Hamburg, Germany) and was propagated in L929 cells as described. Mice were infected intravenously with 2×10^6^ plaque forming units (pfu) LCMV-WE. We observed no severe pathology until mice were sacrificed for analysis and there was no difference in survival between both groups in this experimental setting. Virus titers were measured using a plaque forming assay as described previously [69]. Briefly, organs were harvested into HBSS and homogenized using a Tissue Lyser (Qiagen). 0.8×10^6^ MC57 cells were added to previously in 10-fold dilutions titrated virus samples on 24-well plates. After 3h 1% methylcellulose containing medium was added. After 48 h plates were fixed (4% formalin), permeabilized (1% Triton X HBSS), and stained with anti-VL-4 antibody, peroxidase anti-rat antibody and PPND solved in 50 mM Na_2_HPO_4_ and 25 mM citric acid. Histological analysis was performed on snap frozen tissue as described [69]. Anti-LCMV-NP (clone: VL4) was used in combination with an alkaline phosphatase system. Tetramer production, surface and intracellular FCM staining was performed as described previously [69]. Briefly, single cell suspensions from spleen and liver tissue as well as peripheral blood lymphocytes were stained using gp33 or np396 MHC class I tetramers (gp33/H-2Db) for 15 min or gp61 MHC II tetramer for 30 min at 37°C, followed by staining with anti-CD8 (BD Biosciences) for 30 min at 4°C. For determination of their activation status, lymphocytes were stained with antibodies against surface molecules as indicated for 30 min at 4°C. For intracellular cytokine stain single suspended splenocytes or liver cells were incubated with the LCMV-specific peptides gp33, np396, or gp61. After 1 h Brefeldin A (eBiosciences) was added, followed by additional 5 h incubation at 37°C. After surface stain with anti-CD8 or anti-CD4 (eBiosciences) cells were fixed with 2% formalin and permeabilized with PBS containing 1% FCS and 0.1% Saponin and stained with anti-IFNγ (eBiosciences) for 30 min at 4°C.

### Ethics statement

All mice were maintained under specific pathogen-free conditions and experiments have been approved by the LANUV in accordance with German laws for animal protection (reference number G315).

### Microarray analysis

IFNα-induced gene expression data [4] was analyzed by the Robust Multi-array Average (RMA) [70] algorithm. It was applied for data processing of Affymetrix gene expression data (Human Gene ST Arrays) using the implementation in the simpleaffy R package version 2.40.0 (http://www.bioconductor.org/packages/release/bioc/html/simpleaffy.html). All subsequent analyses were performed on the log_2_-scale and the expression of the individual genes was considered relative to the measured expression of untreated cells at 0 hours. A paired t-test (treated vs. untreated) was used to assess the significance of IFNα-induced regulation at 1, 2, 3, 4, 8, 12, 24 hours. Because only three genes (*ID8139776*, *TCEB3CL2*, *CFC1*) were significantly downregulated, we focused on the 53 genes showing a significant upregulation (p < 0.05 and average fold change > 2). The time point of maximal induction (t_max_) was considered to subdivide the upregulated genes into three classes: early (t_max_ ≤ 4 h), intermediate (t_max_ = 8 h) and late (t_max_ ≥ 12 h). The time point of maximal induction (t_max_) was previously employed to classify the dynamics of gene expression [38]. Because there is no prior knowledge about the time point of maximal induction, a pre-specified null hypothesis cannot be formulated. Therefore, it was not possible to employ statistical tests. Rather, in our setting simply the observed peak time defines the unbiased maximum likelihood estimate. Assigning only two groups (early and late) would be a more stringent decision but would not solve the issue of discriminating reasonably between the clusters. Since we did not see variations in EC_50_ (Fig 2A) and the mRNA stability gradually increased between early, intermediate and late genes (Fig 2B), an alternative assignment would have a minor impact on our results. Genes were visualized with respect to the time-point of maximal regulation and within the groups with the same time-point according to the fold change at 1 h.

### Quantification of RNA stability

Cells were seeded, growth factor depleted and stimulated with 500 U/ml IFNα for 8 hours as described above followed by treatment with 5 μg/ml actinomycin D to inhibit transcription. Total RNA was extracted at specific time points and analyzed using qRT-PCR. RNA half-life was estimated by fitting the mRNA fold expression to an exponential decay 3-parameter function. t_1/2_: mRNA half-life.

$$f(t)=d+a\cdot \exp\left(-\frac{\ln(2)\cdot t}{t_{1/2}}\right)$$

### Quantification of dose-dependency of RNA on IFNα

Cells were seeded, growth factor depleted and stimulated as described above and stimulated with increasing doses of IFNα for 4 hours. Total RNA was extracted and analyzed using qRT-PCR. A sigmoidal 4-parameter Hill function was fitted to the RNA expression.

$$f(x)=d+\frac{a\cdot x^{b}}{c^{b}+x^{b}}$$

Where *d* = y-axis intercept, *a* = amplitude, *b* = slope and *c* = x-value of the point of inflection i.e. the EC_50_ dose.

### Transcription factor binding site analysis

Transcription factor binding site analysis was performed using HOMER software [22] (http://homer.salk.edu/homer/ngs/index.html). Promoter regions of analyzed genes were analyzed for known transcription factor binding sites. For this, a list with identifiers of genes of interest was submitted to the software and the respective promoter regions were obtained from a software-specific database. Significant enrichment of found transcription factor binding sites were set relative to all promoter regions analyzed using hypergeometric test.

### Mathematical modeling

The presented modeling approach is based upon a previously published IFNα model [4]. For this study, the model has been extended by incorporating the genetic response of IFNα-stimulated JAK/STAT signaling. Further, the formation of the receptor complex was simplified so that the complex is activated directly by IFNα binding. In addition, ISGF3 formation and dissociation were previously incorporated as two steps. Here, the complete formation of ISGF3 was summarized in one step; STAT1, STAT2 and IRF9 bind synergistically. The final model consists of 30 species and 53 kinetic parameters. All reactions are defined as ordinary differential equations (ODEs) based on mass action kinetics in cytoplasm and nucleus. Measured concentrations (STAT1, STAT2, IRF9 and IFN) were transformed from molecules per cell to nM by using STAT1 concentration as reference. In the final version of the model, unphosphorylated STAT1 concentration was identified to be negligible. The current model is implemented into the MATLAB-based modeling framework D2D [71, 72].

### Parameter estimation

To find the optimal parameter sets that describe the experimental data for each model structure best, we performed numerical parameter estimation. The D2D framework is using a parallelized implementation of the CVODES ODE solver. The procedure of parameter estimation is based on multiple local optimizations for different initial guesses of the parameters. For the optimization, the LSQNONLIN algorithm (MATLAB, R2011a, Mathworks) was used. Most kinetic parameters were limited to values between 10^−6^ and 1. Exceptions include translocation parameters. Here, the upper boundary was raised to 10^2^. Parameter values close to upper or lower boundaries result from practical non-identifiability of the model structure. We assume that six orders of magnitude as a parameter range is sufficient to not hinder the parameter estimation process. For the random sampling of the multiple starting points, a Latin hypercube method was utilized. In addition to kinetic parameters, the observation function relating the ODE model to the experimentally accessible data contains scaling and noise parameters. These non-kinetic parameters were fitted in parallel to the kinetic parameters as described [73]. Using a previously established strategy [73], we ensured reliable convergence of our parameter estimation procedure for the two mathematical models (S2C Fig).

### Prediction profiles

To obtain confidence intervals of the model predictions for the additional internal feedback loops, we calculated prediction profiles for the respective species as described [74]. For our analysis, prediction profiles have been calculated along the complete time course of the core model with an additional intracellular feedback and species “internal_x_factor_mrna” (Fig 4B). Through the calculation of prediction profiles, a range for the specified trajectories of the species dynamics is given for each calculated time point, in which the likelihood value of the model stays within a 95% confidence level.

### Rankings (AIC/LRT)

Performances of different model structures are determined by the likelihood *L*. For comparison of the model structures, two different criteria are used (S2D and S2E Fig). First, we introduced a variation of the likelihood-ratio test:

$$icdf_{\Delta df}(0.95)-2\cdot \log(L)$$

where *Δdf* denotes the difference in degrees of freedom between the two selected models and *icdf* denotes the inverse cumulative density function of the chi-squared distribution. The results of the likelihood ratio tests with the full model are then used to obtain the ranking of the corresponding model structures.

For the second criterion, all models are compared utilizing the Akaike Information Criterion (AIC), defined as:

AIC=2⋅k−2⋅log(L)

where *k* denotes the degrees of freedom in the respective model. While the AIC provides a ranking where each model is treated equally, the LRT provides information in terms of significance for a pairwise comparison of two selected models. In practice, the AIC slightly favors larger models due to the linear penalization of the degrees of freedom of a model.

## Supporting information

S1 Fig Core mathematical model of IFNα-induced JAK/STAT signaling and gene expression. (**A**) Schematic representation of the core model according to Systems Biology Graphical Notation. TFBS: transcription factor-binding site. (**B**,**C**) Trajectories of the core model are shown together with the dynamic behavior of the core components of the JAK/STAT signaling pathway measured by quantitative immunoblotting (**B**) and to the expression of IFNα-induced genes examined by qRT-PCR (**C**) after stimulation of Huh7.5 cells with 500 U/ml IFNα. pJAK1 and SOCS1 were measured in cytoplasmic lysates after immunoprecipitations, pSTAT1 and USP18 were measured in cytoplasmic lysates and IRF9 was measured in nuclear lysates. Filled circles: experimental data; line: model trajectories, shades: estimated error; a.u. arbitrary units.
(TIF)

S2 Fig The core model with an additional intracellular feedback is superior and gene expression upon downregulation of IRF family members. (**A**) Model rankings according to likelihood ratio test presented by the negative logarithmic likelihood penalized by parameter difference. Lower value indicates preferred model. (**B**) Model rankings according to Akaike information criteria (AIC). The preferred model is the one with the smaller AIC value. (**C**) Assessment of the optimization performance by a waterfall plot. The best parameters were reproducibly found, which validates the applied model calibration approach. (**D**) Huh7.5 cells were growth factor depleted and pre-incubated for 24 hours with siRNA directed against *IRF2*, *IRF4*, *IRF8* or their combinations followed by 500 U/ml IFNα treatment. At indicated time points RNA was extracted and analyzed using qRT-PCR. Error bars represent SD (n = 3). Expression differences at the 24 hour time point were tested by two-sided t-tests using Bonferroni correction (m = 7). *, p<0.05; ***, p<0.001. (**E**) Expression profile of *IRF4* mRNA after treatment with 500 U/ml IFNα was detected by qRT-PCR. (**F**) Expression profile of *IRF8* mRNA after treatment with 500 U/ml IFNα was detected by qRT-PCR. (**G**) Huh7.5 cells were growth factor depleted and pre-incubated for 24 hours with siRNA directed against IRF2 followed by 500 U/ml IFNa treatment. At indicated time points RNA was extracted and analyzed using qRT-PCR. Error bars represent SD (n = 3). (**H**) Gene expression upon decreased *IRF8* expression. Huh7.5 cells were incubated with 50 nM siRNA directed against *IRF8* (red), against *IRF8* and *IRF2* (lilac) or non-targeting control (blue) for 24 hours, and then treated with 500 U/ml IFNα. The cells were lysed at the indicated time points and total RNA was extracted and analyzed by qRT-PCR. The error bars represent SD of biological triplicates.
(TIF)

S3 Fig IFNα-induced gene expression after co-stimulation with IL8 or IL6 and the activation of STAT3 by IL1β is blocked by Stattic. (**A**) Co-stimulation with IFNα and IL8. Huh7.5 cells were growth factor depleted followed by single treatment with 500 U/ml IFNα alone or in combination with 10 ng/ml IL8. At indicated time points RNA was extracted and analyzed using qRT-PCR. Error bars represent SD of biological triplicates. (**B**) Co-stimulation with IFNα and IL6. Huh7.5 cells were growth factor depleted followed by single treatment with 500 U/ml IFNα alone or in combination with 5 ng/ml IL6. At indicated time points RNA was extracted and analyzed using qRT-PCR. Error bars represent SD of biological triplicates. (**C**) Huh7.5 cells were single or co-stimulated with 500 U/ml IFNα and 10 ng/ml IL1β. Cells were lysed at indicated time points and analyzed using quantitative immunoblotting. Error bars represent SEM of three biological replicates. (**D**) Huh7.5 cells were treated with 10 μM Stattic for up to 24 h or left untreated and cell viability was measured. (**E**) Huh7.5 cells were pre-treated with 10 μM Stattic followed by 10 ng/ml IL1β and 500 U/ml IFNα treatment. Cells were lysed at indicated time points and analyzed using quantitative immunoblotting. Error bars represent SD of biological triplicates. a.u.: arbitrary units.
(TIF)

S4 Fig Enhanced viral clearance in a HCV replicon cell line upon co-stimulation with IFNα and IL1β. (**A**) Luciferase activity measurement in single and co-stimulated cells. Time-resolved measurements of luciferase activity in cells treated with 500 U/ml IFNα alone or in combination with 10 ng/ml IL1β compared to the unstimulated control. Error bars represent SEM of three biological replicates. (**B**) Luciferase activity measurement in single and co-stimulated cells. Time-resolved measurements of luciferase activity in cells treated with 50 U/ml IFNα alone or pre-treatment with 50 U/ml IFNα followed by 1 ng/ml IL1β treatment compared to the unstimulated control. Error bars represent SEM of four biological replicates. (**C**) Expression of the selected antiviral genes in primary mouse hepatocytes from wild-type (WT) or IL1R1 knock-out (Il1r1^-/-^) mice upon stimulation with 500 U/ml murine IFNαA, 10 ng/ml murine IL1β or co-treatment. RNA was extracted at the indicated time points and analyzed by qRT-PCR. Error bars represent SEM of four biological replicates; a.u.: arbitrary units. (**D**) Primary murine hepatocytes derived from three mice were analyzed by immunofluorescence using an antibody specific for F4/80 and nuclei were stained with Hoechst 33258. As a control cells were incubated only with the FITC-labeled secondary antibody (2^nd^ Ab ctrl). The ratio of F4/80-positive cells to the total number of cells was calculated for each image and the results are displayed as box plots.
(TIF)

S5 Fig Reduction of anti-viral T cell immunity following LCMV infection in Il1r1^-/-^ mice. (**A**) Wild-type (WT) or Il1r1 knock-out (Il1r1^-/-^) CL57BL/6 mice were infected with 2×10^6^ pfu of LCMV WE. Four and eight days post infection, single cell suspensions from spleen and liver tissue as well as peripheral blood lymphocytes were stained using gp33 or np396 MHC class I tetramers or gp61 MHC II tetramer followed by staining with anti-CD8. Differences between WT and Il1r1^-/-^ cells were tested by two-way ANOVA. ***, p<0.001; **, p<0.01; *, p<0.05; n.s., not significant, n = 6. (**B**) Four and eight days post infection, suspended liver cells or splenocytes were stained with the LCMV-specific peptides gp33, np396, or gp61. Additionally, surface staining with anti-CD8 or anti-CD4 antibodies and intracellular staining with anti-IFNγ antibodies was performed. Differences between WT and Il1r1^-/-^ cells were tested by two-way ANOVA. *, p<0.05; n.s., not significant, n = 6. (**C**) Four and eight days post infection, lymphocytes were stained with antibodies against surface molecules.
(TIF)

S1 Table Initial concentrations of model species. Measured concentrations (JAK1, TYK2, STAT1, STAT2, IRF9) were transformed from molecules per cell to nM by using STAT1 concentration as reference. Concentrations for receptors were assumed to be non-limiting and therefore set to a high amount [4].
(DOCX)

S2 Table qRT-PCR primers and corresponding UPL probes. (DOCX)

S1 Data Numerical data. Excel spreadsheet containing, in separate sheets, the underlying numerical data for figure panels 1C, 1D, 2A, 2B, 4C, 4D, 5A, 5B, 6A, 6B, 6C, 6D, 7A, 7C, 8A, 8B, 8C, S2D, S2E, S2F, S2G, S2H, S3A, S3B, S3C, S3D, S3E, S4A, S4B, S4C, S4D, S5A and S5B.
(XLSX)
