# Supplementary figures and images for: Identification of Interleukin1β as an Amplifier of Interferon alpha-induced Antiviral Responses
Source: PLoS Pathog. 2020 Oct 1;16(10):e1008461. doi: 10.1371/journal.ppat.1008461 (PMC7553310; doi:10.1371/journal.ppat.1008461)

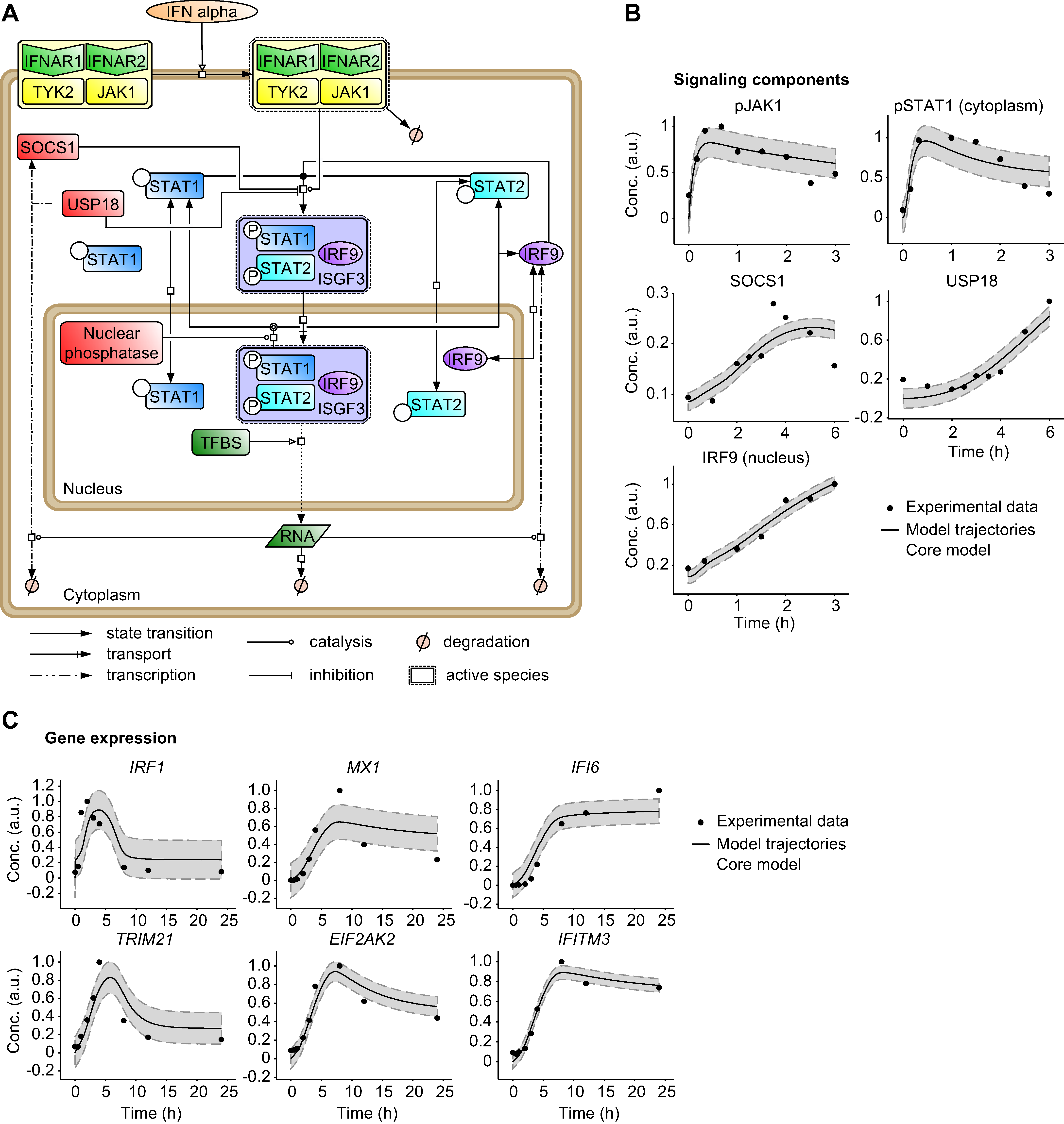

Supplement: S1 Fig — (A) Schematic representation of the core model according to Systems Biology Graphical Notation. TFBS: transcription factor-binding site. (B,C) Trajectories of the core model are shown together with the dynamic behavior of the core components of the JAK/STAT signaling pathway measured by quantitative immunoblotting (B) and to the expression of IFNα-induced genes examined by qRT-PCR (C) after stimulation of Huh7.5 cells with 500 U/ml IFNα. pJAK1 and SOCS1 were measured in cytoplasmic lysates after immunoprecipitations, pSTAT1 and USP18 were measured in cytoplasmic lysates and IRF9 was measured in nuclear lysates. Filled circles: experimental data; line: model trajectories, shades: estimated error; a.u. arbitrary units. (TIF) [file ppat.1008461.s001.tif]

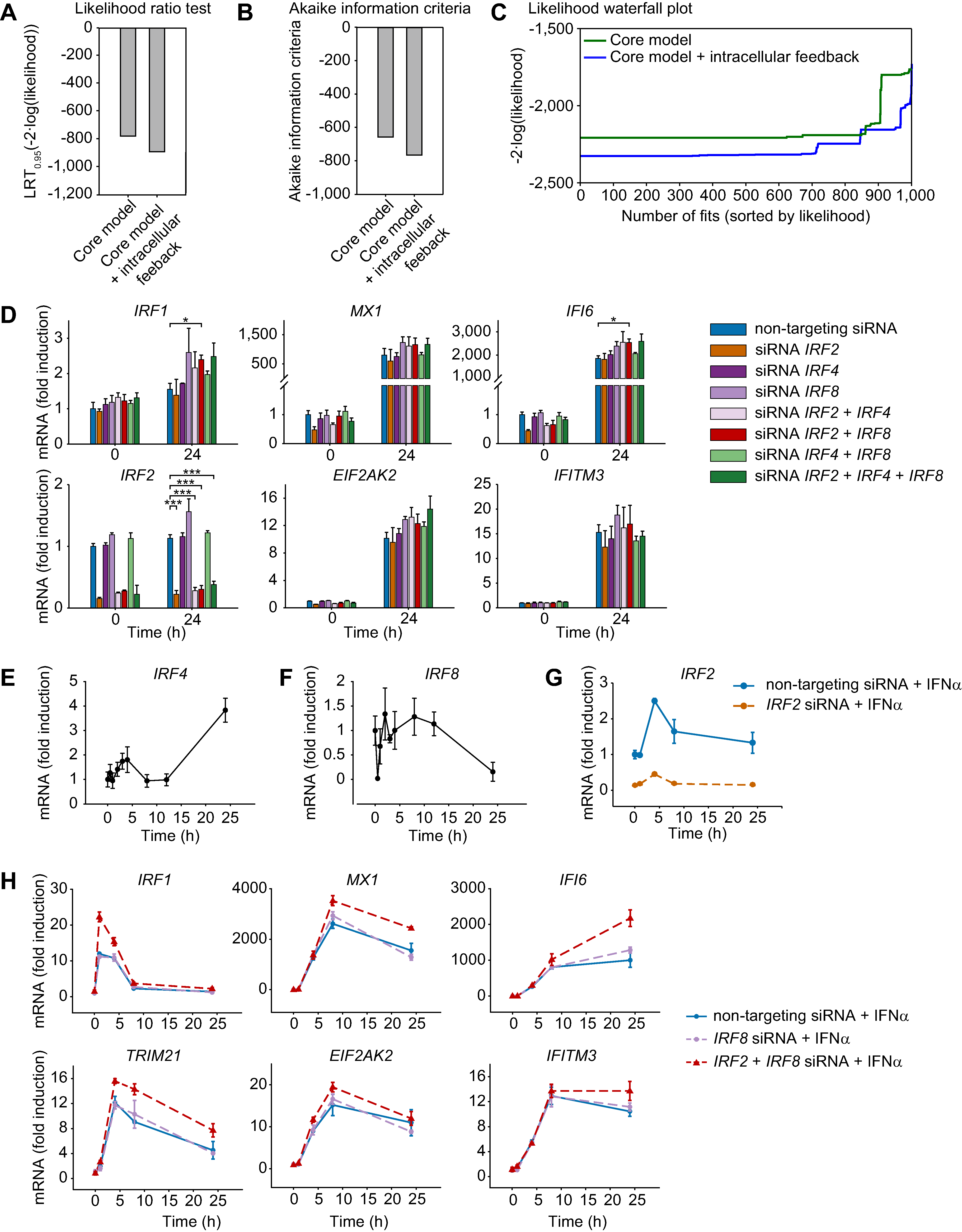

Supplement: S2 Fig — (A) Model rankings according to likelihood ratio test presented by the negative logarithmic likelihood penalized by parameter difference. Lower value indicates preferred model. (B) Model rankings according to Akaike information criteria (AIC). The preferred model is the one with the smaller AIC value. (C) Assessment of the optimization performance by a waterfall plot. The best parameters were reproducibly found, which validates the applied model calibration approach. (D) Huh7.5 cells were growth factor depleted and pre-incubated for 24 hours with siRNA directed against IRF2, IRF4, IRF8 or their combinations followed by 500 U/ml IFNα treatment. At indicated time points RNA was extracted and analyzed using qRT-PCR. Error bars represent SD (n = 3). Expression differences at the 24 hour time point were tested by two-sided t-tests using Bonferroni correction (m = 7). *, p<0.05; ***, p<0.001. (E) Expression profile of IRF4 mRNA after treatment with 500 U/ml IFNα was detected by qRT-PCR. (F) Expression profile of IRF8 mRNA after treatment with 500 U/ml IFNα was detected by qRT-PCR. (G) Huh7.5 cells were growth factor depleted and pre-incubated for 24 hours with siRNA directed against IRF2 followed by 500 U/ml IFNa treatment. At indicated time points RNA was extracted and analyzed using qRT-PCR. Error bars represent SD (n = 3). (H) Gene expression upon decreased IRF8 expression. Huh7.5 cells were incubated with 50 nM siRNA directed against IRF8 (red), against IRF8 and IRF2 (lilac) or non-targeting control (blue) for 24 hours, and then treated with 500 U/ml IFNα. The cells were lysed at the indicated time points and total RNA was extracted and analyzed by qRT-PCR. The error bars represent SD of biological triplicates. (TIF) [file ppat.1008461.s002.tif]

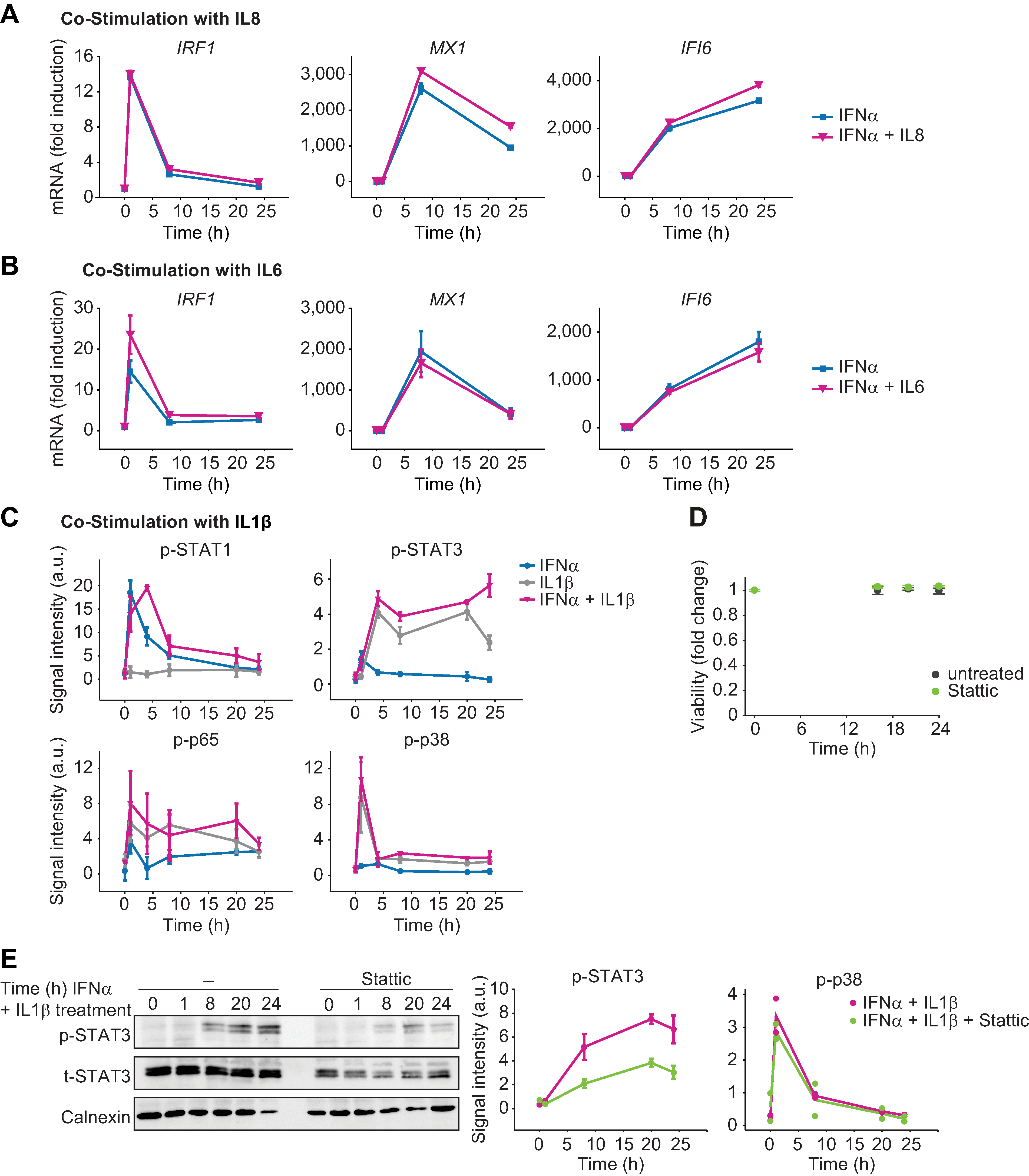

Supplement: S3 Fig — (A) Co-stimulation with IFNα and IL8. Huh7.5 cells were growth factor depleted followed by single treatment with 500 U/ml IFNα alone or in combination with 10 ng/ml IL8. At indicated time points RNA was extracted and analyzed using qRT-PCR. Error bars represent SD of biological triplicates. (B) Co-stimulation with IFNα and IL6. Huh7.5 cells were growth factor depleted followed by single treatment with 500 U/ml IFNα alone or in combination with 5 ng/ml IL6. At indicated time points RNA was extracted and analyzed using qRT-PCR. Error bars represent SD of biological triplicates. (C) Huh7.5 cells were single or co-stimulated with 500 U/ml IFNα and 10 ng/ml IL1β. Cells were lysed at indicated time points and analyzed using quantitative immunoblotting. Error bars represent SEM of three biological replicates. (D) Huh7.5 cells were treated with 10 μM Stattic for up to 24 h or left untreated and cell viability was measured. (E) Huh7.5 cells were pre-treated with 10 μM Stattic followed by 10 ng/ml IL1β and 500 U/ml IFNα treatment. Cells were lysed at indicated time points and analyzed using quantitative immunoblotting. Error bars represent SD of biological triplicates. a.u.: arbitrary units. (TIF) [file ppat.1008461.s003.tif]

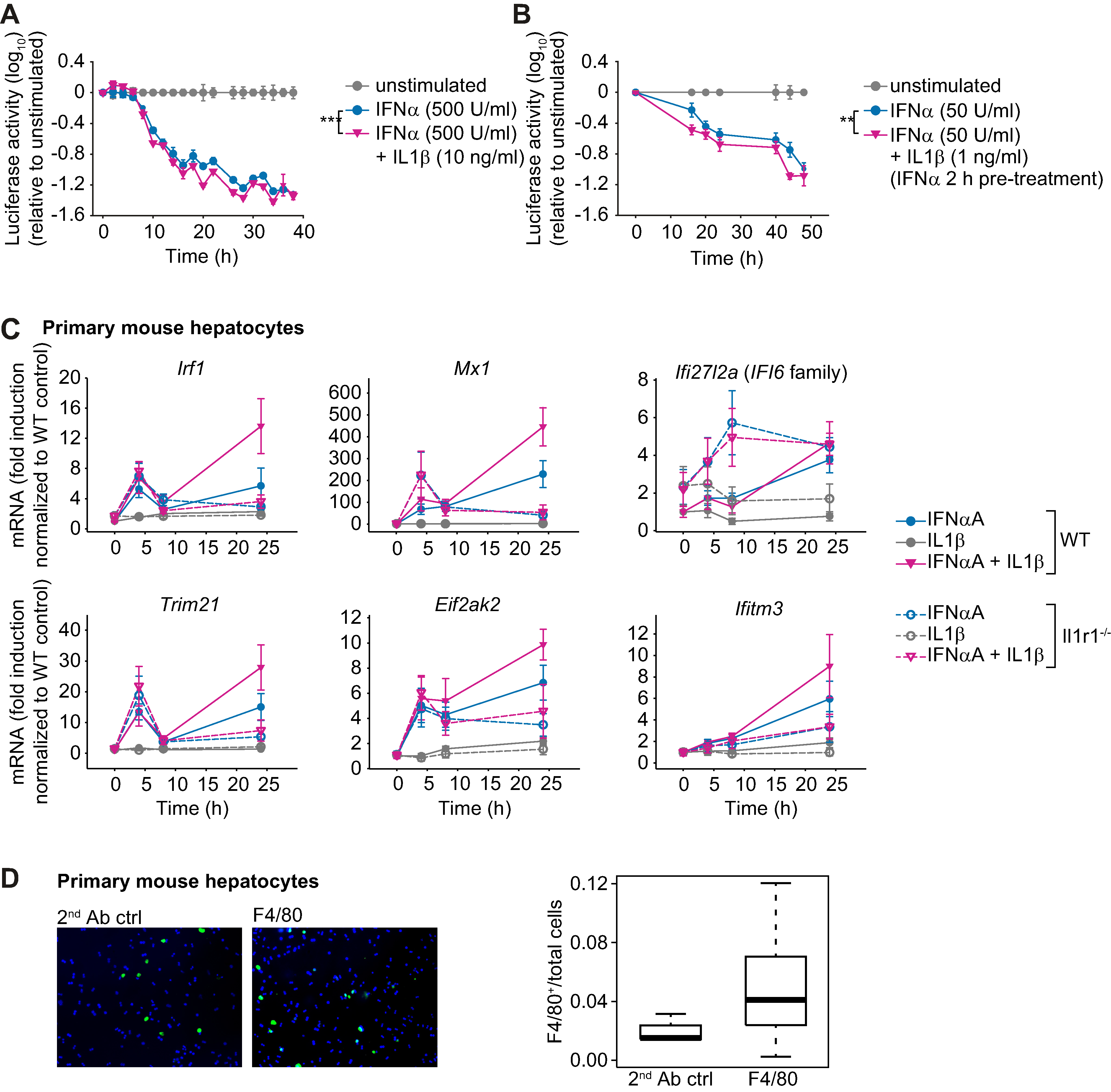

Supplement: S4 Fig — (A) Luciferase activity measurement in single and co-stimulated cells. Time-resolved measurements of luciferase activity in cells treated with 500 U/ml IFNα alone or in combination with 10 ng/ml IL1β compared to the unstimulated control. Error bars represent SEM of three biological replicates. (B) Luciferase activity measurement in single and co-stimulated cells. Time-resolved measurements of luciferase activity in cells treated with 50 U/ml IFNα alone or pre-treatment with 50 U/ml IFNα followed by 1 ng/ml IL1β treatment compared to the unstimulated control. Error bars represent SEM of four biological replicates. (C) Expression of the selected antiviral genes in primary mouse hepatocytes from wild-type (WT) or IL1R1 knock-out (Il1r1-/-) mice upon stimulation with 500 U/ml murine IFNαA, 10 ng/ml murine IL1β or co-treatment. RNA was extracted at the indicated time points and analyzed by qRT-PCR. Error bars represent SEM of four biological replicates; a.u.: arbitrary units. (D) Primary murine hepatocytes derived from three mice were analyzed by immunofluorescence using an antibody specific for F4/80 and nuclei were stained with Hoechst 33258. As a control cells were incubated only with the FITC-labeled secondary antibody (2nd Ab ctrl). The ratio of F4/80-positive cells to the total number of cells was calculated for each image and the results are displayed as box plots. (TIF) [file ppat.1008461.s004.tif]

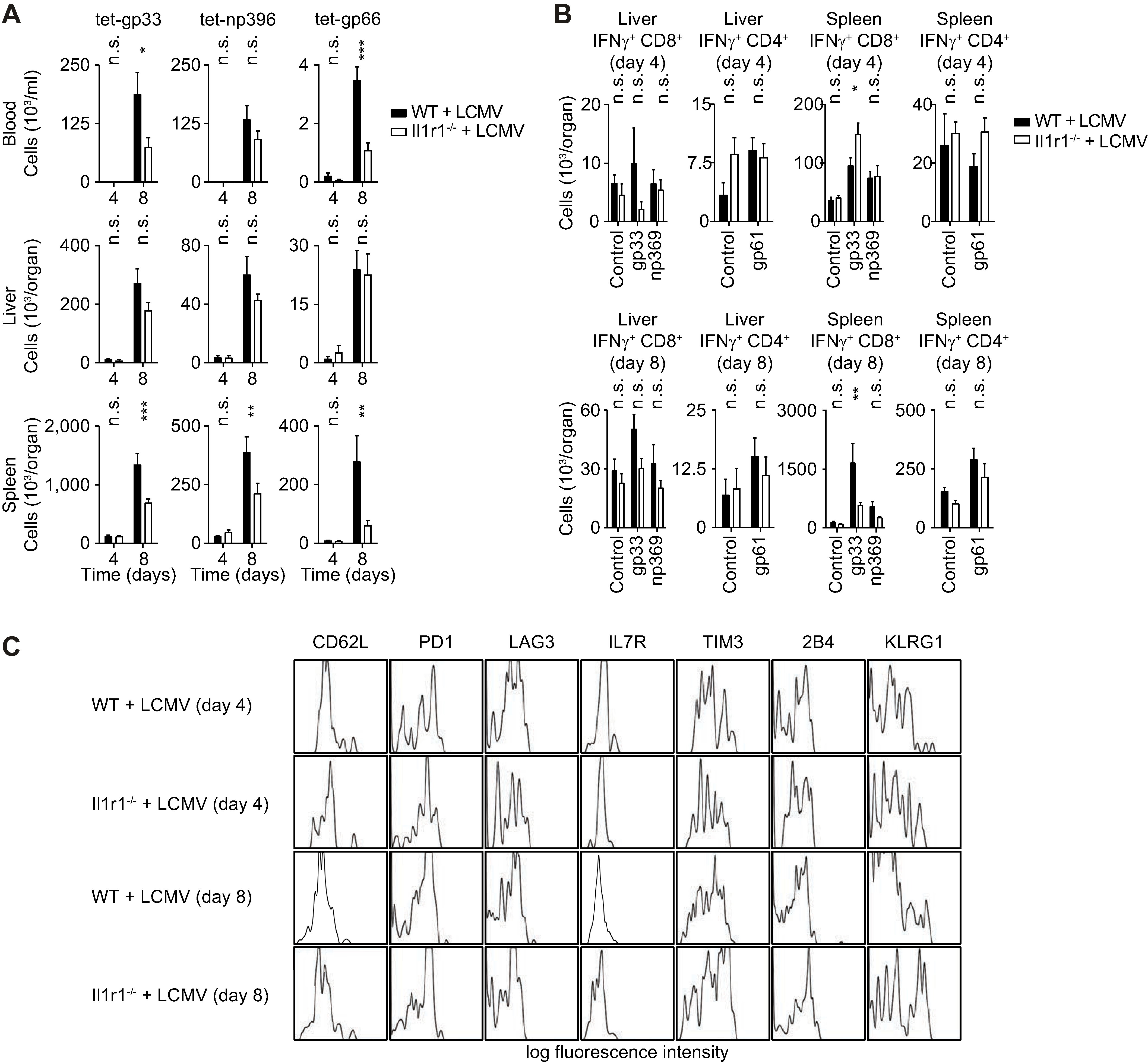

Supplement: S5 Fig — (A) Wild-type (WT) or Il1r1 knock-out (Il1r1-/-) CL57BL/6 mice were infected with 2×10^6^ pfu of LCMV WE. Four and eight days post infection, single cell suspensions from spleen and liver tissue as well as peripheral blood lymphocytes were stained using gp33 or np396 MHC class I tetramers or gp61 MHC II tetramer followed by staining with anti-CD8. Differences between WT and Il1r1-/- cells were tested by two-way ANOVA. ***, p<0.001; **, p<0.01; *, p<0.05; n.s., not significant, n = 6. (B) Four and eight days post infection, suspended liver cells or splenocytes were stained with the LCMV-specific peptides gp33, np396, or gp61. Additionally, surface staining with anti-CD8 or anti-CD4 antibodies and intracellular staining with anti-IFNγ antibodies was performed. Differences between WT and Il1r1-/- cells were tested by two-way ANOVA. *, p<0.05; n.s., not significant, n = 6. (C) Four and eight days post infection, lymphocytes were stained with antibodies against surface molecules. (TIF) [file ppat.1008461.s005.tif]
