# Supplementary material for: Identification of Interleukin1β as an Amplifier of Interferon alpha-induced Antiviral Responses
Source: PLoS Pathog. 2020 Oct 1;16(10):e1008461. doi: 10.1371/journal.ppat.1008461 (PMC7553310; doi:10.1371/journal.ppat.1008461)
Supplement: S1 Table — Measured concentrations (JAK1, TYK2, STAT1, STAT2, IRF9) were transformed from molecules per cell to nM by using STAT1 concentration as reference. Concentrations for receptors were assumed to be non-limiting and therefore set to a high amount [4]. (DOCX) [file ppat.1008461.s006.docx]

**S1 Table**

| Species | Initial concentrations (nM) |
| --- | --- |
| IFNAR1 | 1000 nM |
| IFNAR2 | 1000 nM |
| JAK | 26 nM |
| TYK | 13 nM |
| IFN | 6.5 nM |
| STAT1 | 1500 nM |
| STAT2 | 500 nM |
| IRF9 | 45 nM |
| CP | 20 nM |
| NP | 40 nM |
| PIAS | 10 nM |
| TFBS | 500 nM |
