# Supplementary material for: Identification of Interleukin1β as an Amplifier of Interferon alpha-induced Antiviral Responses
Source: PLoS Pathog. 2020 Oct 1;16(10):e1008461. doi: 10.1371/journal.ppat.1008461 (PMC7553310; doi:10.1371/journal.ppat.1008461)
Supplement: S2 Table — (DOCX) [file ppat.1008461.s007.docx]

S2 Table.

| Gene | Species | Accession # | UPL Probe # | Primer Forward | Primer Reverse |
| --- | --- | --- | --- | --- | --- |
| *HPRT* | human | NM_000194 | 73 | cgagcaagacgttcagtcct | tgaccttgatttattttgcatacc |
| *IFI6* | human | NM_002038 | 40 | gggctccgtcactagacctt | aaccgtttactcgctgctgt |
| *IFITM3* | human | NM_021034 | 76 | gatgtggatcacggtggac | agatgctcaaggaggagcac |
| *IRF1* | human | NM_002198 | 36 | ttggccttccacgtcttg | gagctgggccattcacac |
| *IRF2* | human | NM_002199 | 56 | tgaagtggatagtacggtgaaca | cggattggtgacaatctcttg |
| *IRF4* | human | NM_002460 | 55 | gccaagattccaggtgactc | ctggctagcagaggttctacg |
| *IRF8* | human | NM_002163 | 20 | gaggtggtccaggtcttcg | cggccctggctgttatag |
| *IRF9* | human | NM_006084 | 77 | aactgcccactctccacttg | agcctggacagcaactcag |
| *ISG15* | human | NM_005101 | 24 | ggcttgaggccgtactcc | ctgttctggctgaccttcg |
| *ISG56* | human | NM_001548 | 9 | gctccagactatccttgacctg | agaacggctgcctaatttacag |
| *MX1* | human | NM_002462 | 42 | gagctgttctcctgcacctc | ctcccactccctgaaatctg |
| *EIF2AK2* | human | NM_002759 | 62 | cggtatgtattaagttcctccatga | gacaaagcttccaaccagga |
| *SOCS1* | human | NM_003955 | 87 | gccccttctgtaggatggta | ctgctgtggagactgcattg |
| *SOCS3* | human | NM_002759 | 55 | gcgaggatcctggtgaca | ccaggatggttcccttcag |
| *TRIM21* | human | NM_003141 | 24 | tggagacctttagggggttt | tgagcggaaactgaaagtga |
| *USP18* | human | NM_017414 | 75 | tatgtgagccaggcacgat | tcccgacgtggaactcag |
| *Hprt* | mouse | NM_000194 | 73 | tatgccgaggatttggaaaaagtg | acagagggccacaatgtgatg |
| *Ifitm3* | mouse | NM_021034 | 76 | gatcaacatgcccagagaggt | catcacccaccatcttccgat |
| *Irf1* | mouse | NM_002198 | 36 | tcgaatgcggatgagaccct | gcttagcagcgtgcttccat |
| *Isg12* | mouse | NM_002038 | 40 | tgccaatggaggtggagttg | agcccctaggatggcatttg |
| *Mx1* | mouse | NM_002462 | 42 | tggaagcactgtctggagtg | gcctctccactcctctcctt |
| *Eif2ak2* | mouse | NM_002759 | 62 | cttcgggacctccacatgac | gctaatttggctgcagcgtt |
| *Ro52* | mouse | NM_003141 | 24 | agtctgggaaacaccgggac | tcggccaactctttcccctt |
